# Analysis of genes within the schizophrenia-linked 22q11.2 deletion identifies interaction of *night owl/LZTR1* and *NF1* in GABAergic sleep control

**DOI:** 10.1371/journal.pgen.1008727

**Published:** 2020-04-27

**Authors:** Gianna W. Maurer, Alina Malita, Stanislav Nagy, Takashi Koyama, Thomas M. Werge, Kenneth A. Halberg, Michael J. Texada, Kim Rewitz

**Affiliations:** 1 Department of Biology, University of Copenhagen, Copenhagen, Denmark; 2 Institute for Biological Psychiatry, Mental Health Centre Sct. Hans, Roskilde, Denmark; Biocenter, Universität Würzburg, GERMANY

## Abstract

The human 22q11.2 chromosomal deletion is one of the strongest identified genetic risk factors for schizophrenia. Although the deletion spans a number of known genes, the contribution of each of these to the 22q11.2 deletion syndrome (DS) is not known. To investigate the effect of individual genes within this interval on the pathophysiology associated with the deletion, we analyzed their role in sleep, a behavior affected in virtually all psychiatric disorders, including the 22q11.2 DS. We identified the gene *LZTR1* (*night owl*, *nowl*) as a regulator of night-time sleep in *Drosophila*. In humans, *LZTR1* has been associated with Ras-dependent neurological diseases also caused by *Neurofibromin-1* (*Nf1*) deficiency. We show that *Nf1* loss leads to a night-time sleep phenotype nearly identical to that of *nowl* loss and that *nowl* negatively regulates Ras and interacts with *Nf1* in sleep regulation. Furthermore, *nowl* is required for metabolic homeostasis, suggesting that *LZTR1* may contribute to the genetic susceptibility to obesity associated with the 22q11.2 DS. Knockdown of *nowl* or *Nf1* in GABA-responsive sleep-promoting neurons elicits the sleep phenotype, and this defect can be rescued by increased GABA_A_ receptor signaling, indicating that Nowl regulates sleep through modulation of GABA signaling. Our results suggest that *nowl*/*LZTR1* may be a conserved regulator of GABA signaling important for normal sleep that contributes to the 22q11.2 DS.

## Author summary

Schizophrenia is a devastating mental disorder with a large genetic component to disease predisposition. One of the strongest genetic risk factors for this disorder is a relatively small genetic deletion of 43 genes on the 22^nd^ chromosome, called 22q11.2, which confers about a 25% risk of schizophrenia development. However, it is likely that only some of these deleted genes affect disease risk, so we tested most of them individually. One of the main symptoms of schizophrenia is disturbed sleep. Sleep is an evolutionarily conserved behavior that can be easily studied in the fruit fly *Drosophila melanogaster*, so we investigated the effect on sleep of blocking expression of the fly homologs of most of the 22q11.2 genes and identified the gene *LZTR1* (*night owl*, *nowl*) as an important sleep regulator. We found that Nowl/LZTR1 is required for inhibition of the Ras pathway and interacts genetically with the Ras inhibitor NF1. Nowl/LZTR1 appears to function in sleep by modulating inhibitory GABA signaling, which is affected in schizophrenia. Thus, this gene may underlie some of the phenotypes of the human schizophrenia-risk deletion.

## Introduction

Recent genome-wide association studies have identified chromosomal deletions and duplications that confer elevated risk of neuropsychiatric disorders [1, 2]. One of these, the 22q11.2 deletion, which spans 43 genes and occurs in 1 of every 2–4,000 births worldwide [3], is the most prominent known genetic risk factor for development of schizophrenia and is associated with high risk of neuropsychiatric disease [4, 5]. This deletion leads to a variety of phenotypes, including clinical manifestations such as congenital defects of the palate and heart, learning and cognitive disabilities, and sleep disturbances [6]. Sleep abnormalities are among the most common manifestations of neuropsychiatric disease [7], with up to 80% of autism and schizophrenia patients experiencing sleep disturbances [8, 9]. Individuals affected by the 22q11.2 deletion have a roughly 25–30% chance of developing schizophrenia or other psychiatric disorders that include sleep disturbances [10, 11]. However, the possible contribution of each of the individual genes spanned by this deletion to this array of symptoms is not clear. Although certain disease phenotypes have been linked to single genes, such as *TBX1*, found to account for cardiac defects observed in 22q11.2 deletion carriers [12], the genes contributing to the 22q11.2-linked behavioral phenotypes such as sleep disturbance are not characterized.

Many schizophrenic patients exhibit abnormal sleep patterns such as delayed sleep onset, difficulty in maintaining sleep, and a reduced overall amount of sleep [9]. Thus, genes that might play a role in schizophrenia and other behavioral symptoms of the 22q11.2 deletion syndrome (DS) can potentially be identified by their sleep phenotypes. Sleep is an evolutionarily ancient conserved behavior that can be studied in organisms such as the fruit fly *Drosophila melanogaster*, a commonly used invertebrate model. *Drosophila* sleep has been rigorously examined and found to exhibit several of the mammalian hallmarks of sleep, such as sustained periods of quiescence, increased arousal threshold, and circadian and homeostatic regulation [13, 14]. Indeed, recent studies in *Drosophila* have identified genes associated with human neurodevelopmental and psychiatric disorders that disrupt sleep and circadian rhythm, such as the candidate autism-spectrum-disorder gene *Cullin-3* (*Cul3*) [15, 16] and the gene *alicorn* (*alc*) linked to the 1q21.1 deletion, another deletion that confers schizophrenia risk [17].

To identify genes that contribute to the behavioral deficits of the 22q11.2 DS, we examined the role in sleep of individual genes within this interval by knockdown of their *Drosophil*a homologs in the nervous system of the fly. We identified *night owl* (*nowl*, *Lztr1*, *CG3711*), an ortholog of the human *LZTR1* (*leucine-zipper-like transcription regulator 1*), as a modulator of night-time sleep in *Drosophila*. In humans, mutations in *LZTR1* cause neurofibromatosis, a genetic disorder characterized by the growth of benign nervous-system tumors, that is most commonly known to result from mutations in *Neurofibromin-1* (*Nf1*) [18, 19]. Here, we show that neuronal knockdown or mutation of *nowl* causes sleep disturbances including highly increased fragmentation of sleep and reduced total night-time sleep, similar to neuronal loss of *Neurofibromin-1* (*Nf1*) in *Drosophila*. Nf1 is a negative regulator of the Ras signaling pathway and an activator of cAMP signaling [20, 21]. On the other hand, little is known about the function of *LZTR1*, except that its loss has been associated with neurofibromatosis. Interestingly, we find that *nowl* and *Nf1* inhibit Ras signaling and interact genetically in the regulation of sleep in *Drosophila*. Loss of *nowl* affects metabolism and may contribute to the obesity risk associated with the 22q11.2 DS. We find that *nowl* acts through gamma-aminobutyric acid (GABA)-responsive neurons expressing the ionotropic GABA receptor Rdl to maintain sleep. Furthermore, the sleep fragmentation exhibited by *nowl* mutant flies can be rescued by increased inhibitory signaling through this receptor. Because *nowl* knockdown phenocopies the sleep disturbances observed with neuronal *Cul3* knockdown, we suggest that Nowl may interact with Cul3 and Nf1 in GABA-responsive neurons to regulate sleep. Thus, we have identified the 22q11.2-linked gene *nowl*, orthologous to human *LZTR1*, as a regulator of GABAergic signaling and night-time sleep in *Drosophila*. We suggest that *LZTR1* is a promising candidate gene that may contribute to the sleep disturbances and behavioral symptoms seen in patients carrying the 22q11.2 deletion.

## Results

### *Drosophila* screening identifies an ortholog of human *LZTR1* as a regulator of sleep

**Fig 1:**
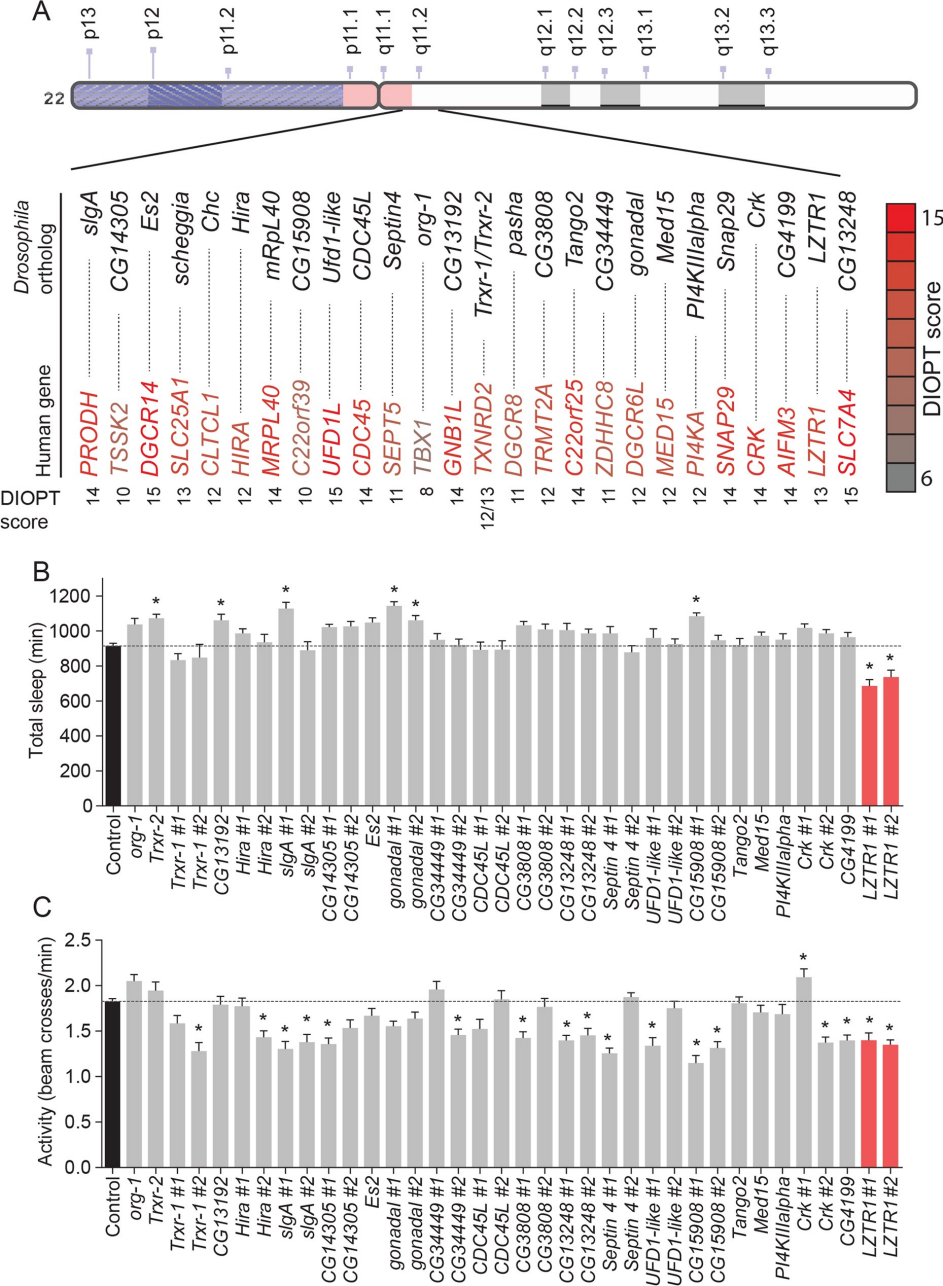
Screening *Drosophila* orthologs of human genes within the 22q11.2 deletion identifies *LZTR1* as a modulator of sleep and activity in *Drosophila*. (A) Schematic diagram of the human 22q11.2 deletion. For the 43 genes spanned by the 22q11.2 deletion, 26 orthologs were identified in *Drosophila melanogaster* with a DIOPT conservation score ≥ 6. (B, C) Total sleep (B) and activity (C) in adult males with pan-neuronal *RNAi*-mediated knockdown of each of these *Drosophila* orthologs. The *elav-GAL4* (*elav>*) pan-neuronal driver line was crossed to *UAS-RNAi* transgenes targeting individual gene orthologs, with n = 16 flies for each genotype. For controls, *elav>* was crossed to *w*^*1118*^, the genetic background for the RNAi lines, with n = 140 flies. When possible, two independent *UAS-RNAi* transgenes were used against each gene. Total sleep (minutes) of 3-to-7-day-old males was recorded over a 24-hour period. Transformant ID numbers for RNAi lines are listed in S1 Table. Genes for which knockdown caused lethality or a non-inflating-wing phenotype were not analyzed. Average activity was measured as the number of beam crosses per minute. Knockdown of *LZTR1* in the nervous system reduces both total sleep and locomotor activity. Graphs represent means with SEM of data pooled from one to five independent experiments. Significance was determined using Kruskal-Wallis test with Dunn's post-hoc testing (* p<0.05).

Sleep disturbance is a common manifestation of neuropsychiatric disorders in general and of the 22q11.2 DS. To determine which, if any, of the individual genes within this deletion might be required for normal sleep, we first identified the *Drosophila* orthologs of the human genes within the 22q11.2 interval. From the 43 genes lying within the 22q11.2 deletion, we identified 26 highly conserved orthologs in *Drosophila* (with a DIOPT score ≥ 6) [22] (Fig 1A). Next, we conducted an RNA-interference (RNAi)-based screen to investigate whether the neuronal function of these *Drosophila* genes might be important for sleep, using the *Drosophila* Activity Monitor (DAM) system (TriKinetics), an automated beam-crossing locomotion assay that is the widely accepted standard for *Drosophila* sleep studies. In these assays, locomotor quiescence of longer than 5 minutes is taken to indicate a sleep-like state, comparable to mammalian sleep [13, 14]. To determine whether each identified *Drosophila* ortholog might play a role in sleep, we knocked each gene down using the nervous system-specific *elav-GAL4* (*elav>*) line to drive RNAi expression. When possible, two independent RNAi lines targeting each gene were tested. The effectiveness of RNAi knockdown was enhanced by the co-expression of *Dicer-2* (*Dcr-2*) [23]. Some RNAi constructs induced lethality when expressed pan-neuronally or displayed a non-inflating wing phenotype due to a gene disruption at the insertion site of the transgene [24]. These constructs were excluded from further analysis.

Pan-neuronal knockdown of several of the *Drosophila* 22q11.2 gene orthologs caused changes in total sleep and activity in adult males (Fig 1B and 1C). Most of these manipulations led to a decrease in locomotor activity, with unchanged or increased sleep amount. Nervous system-specific knockdown of *CG15908* (*C22orf39*), *gonadal* (*DGCR6L*), or *slgA* (*PRODH*) increased the total amount of sleep exhibited by males, while only the knockdown of *CG3711*, the *Drosophila* ortholog of *LZTR1*, caused a decrease in total sleep (Fig 1A). In contrast, in females, neuronal knockdown of several genes led to decreased sleep, including *CDC45L* (human *CDC45*), *CG13192* (*GNB1L*), *Es2* (*DGCR14*), and *Septin4* (*SEPT5*) (S1A Fig). Locomotor activity was affected upon knockdown of several 22q11.2 genes, with most leading to hypoactive phenotypes in both males and females (Fig 1C and S1B Fig). We decided to focus our further studies on *LZTR1*, the only tested gene whose knockdown led to reduced total sleep in combination with decreased locomotor activity in males.

### Neuronal *nowl* function is required for night-time sleep

**Fig 2:**
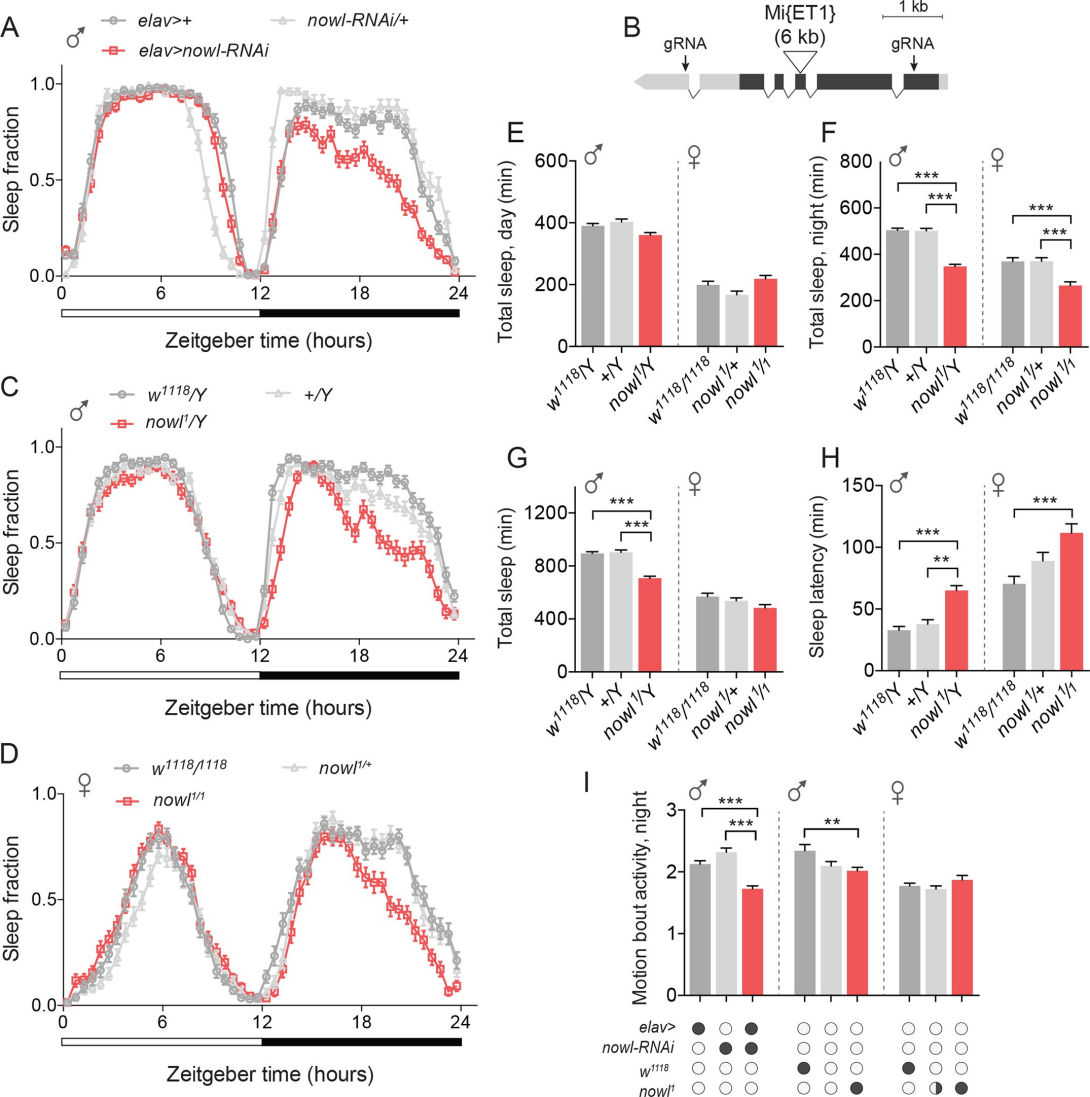
Neuronal loss of the *LZTR1* homolog *night owl* (*nowl)* reduces night-time sleep in *Drosophila*. (A) Daily sleep profiles across a 12-hour light, 12-hour dark (white and black bars) cycle for controls (*elav>+* and *nowl-RNAi/+*) compared to *elav>nowl-RNAi*. Sleep data are binned into 30-minute intervals. (B) Schematic of the *nowl* locus. The gene comprises 5′ and 3′ untranslated regions (light grey) and a multi-exon coding region (dark grey). The locations of the 6-kb *Mi{ET1} Minos* transposable element inserted in the *nowl*^*1*^ allele and the guide-RNA (gRNA) target sites used to induce the *nowl*^*KO*^ CRISPR/Cas9-mediated lesion are indicated. (C, D) Sleep profiles for (C) male *nowl*^*1*^*/Y* and (D) female *nowl*^*1*^*/nowl*^*1*^ mutant flies, compared to control genotypes [in C: *w*^*1118*^ *nowl*^*+*^*/Y* (*w*^*1118*^*/Y*) and *w*^*+*^ *nowl*^*+*^*/Y* (*+/Y*); in D: *w*^*1118/1118*^ and *nowl*^*1*^*/+*]. (E-F) Total day- and night-time sleep (minutes) in male and female flies. (E) Total daytime sleep in male and female *nowl*^*1*^ mutant flies. (F) Total nighttime sleep is significantly decreased in hemizygous male and homozygous female *nowl*^*1*^ mutants, but not in heterozygous females. (G) Overall total sleep (min.) in male and female *nowl*^*1*^ mutant flies compared to controls. Loss of *nowl* in male flies significantly decreases total sleep compared to controls. (H) Sleep latency measured for male and female *nowl*^*1*^ mutant flies compared to controls. Loss of *nowl* in both male and female flies significantly increases sleep latency compared to controls. (I) Quantification of motion-bout activity (min.) during night. Motion-bout activity during the night is significantly reduced in *nowl*-knockdown flies. Graphs represent means with SEM (n = 32–92) of data pooled from one to three independent experiments. Significance was determined using Kruskal-Wallis test with Dunn's post-hoc testing (* p<0.05, ** p<0.01, *** p<0.001).

To investigate the role of *LZTR1* in sleep, we examined the sleep patterns of animals with neuronal knockdown of *LZTR1* (the *LZTR1 #1* RNAi line was used for further experiments; see S1 Table). In light of the reduced-night-time-sleep phenotype displayed by these animals, we chose to refer to this gene as *night owl* (*nowl*) (Fig 2A). We found that nervous-system-specific knockdown of *nowl* (*elav>nowl-RNAi*) reduced night-time sleep compared to controls [*elav>+* and *UAS-nowl-RNAi (nowl-RNAi)/+*, *i*.*e*., *elav>* and *nowl-RNAi* lines crossed to the *w*^*1118*^ genetic background for the RNAi line]. To further investigate the effect of *nowl* loss, we examined the sleep phenotype of a homozygous-viable transposon-insertion mutant of *nowl*, *Mi{ET1}CG3711*^*MB12128*^ (for simplicity we hereafter refer to this allele as *nowl*^*1*^), which carries a 6-kb insertion into the third exon of *nowl* [25] (Fig 2B–2D). Transcript levels of *nowl* have previously been shown to be drastically reduced in this mutant [26]. Like animals with neuronal knockdown, *nowl*^*1*^ mutants exhibited decreased sleep, primarily during the second part of the night, compared with the genetic-background controls [*w*^*1118*^*/Y* and *+/Y*, where “*w*^*1118*^” denotes an X chromosome with a *w*^*-*^ mutation (the genetic background for most *Drosophila* lines), “+” a wild-type X chromosome carrying *w*^*+*^, and “Y” a wild-type Y chromosome]. When sleep data were binned into day and night periods, it showed that *nowl* loss-of-function had a much larger effect on sleep during the night than during the day (Fig 2E–2G). Night-time sleep dropped dramatically in both males and females. Loss of one copy of *nowl* (on the X chromosome) in females did not elicit a phenotype, suggesting that the *nowl*^*1*^ mutation is a recessive loss-of-function allele, consistent with the transcript-level defect previously reported [26]. To further confirm these results, we generated a CRISPR/Cas9-mediated deletion of the *nowl* coding sequence. Like neuronal knockdown and the *nowl*^*1*^ mutation, this deletion mutant (*nowl*^*KO*^) showed reduced night-time sleep (S2A–S2C Fig). Taken together, these data indicate that *nowl* is important for normal sleep behavior, mainly during the night.

Although we observed a decrease in sleep levels overall, sleep levels in these flies still dropped in anticipation of light-dark and dark-light transitions, consistent with an intact circadian clock. To investigate whether *nowl* mutant males do maintain a normal circadian rhythm, adult animals entrained to a 12/12-hour light-dark cycle were kept under constant darkness (free-running conditions) for 8 days and analyzed from 2 h after lights-off transition for 6 days. The average circadian period length of *nowl*^*1*^ mutants during these days was 23.5 hours, which is very similar to the corresponding controls’ free-running periods of 23.6 and 23.5 hours (S3A and S3B Fig). This indicates that the *nowl* mutants can entrain and maintain a normal circadian rhythm with a period similar to the controls. However, when looking at the strength of the circadian clock, we found a reduction in total percentage of rhythmic flies and reduced strength of rhythmicity in flies lacking *nowl* in the nervous system (S3C and S3D Fig). This indicates that these animals have a weaker circadian rhythm, although the *nowl* mutants can maintain a normal period.

### Loss of *nowl* disrupts sleep architecture and phenocopies lack of *Cul3* and *Nf1*

Sleep-onset difficulties are common in psychiatric disorders [27]. Interestingly, male and female *nowl* mutants exhibit increased sleep-onset latency, defined as the time between lights-off and the first sleep bout; that is, they take longer to fall asleep than controls (Fig 2H and S2D Fig). Control males’ latency was 40 minutes, while hemizygous *nowl* mutants on average took 70 minutes to fall asleep after lights-off. Homozygous *nowl-*mutant females also exhibited delayed sleep onset and on average remained active for 110 minutes before their first sleep episode, while control females fell asleep after 70 minutes. However, no sleep-latency phenotype was observed in animals with RNAi-mediated knockdown of *nowl* in the nervous system, suggesting that maybe stronger loss of *nowl* function than provided by RNAi is required to affect sleep onset (Fig 2A).

To confirm that the observed phenotype was related to sleep *per se* and not to altered activity patterns, we analyzed average activity during periods in which the animals were active in the night. Motion-bout activity was significantly reduced when *nowl* was knocked down in the nervous system compared to driver- and *UAS*-alone control genotypes (Fig 2I), while *nowl* mutant flies also displayed reduced or unchanged motion-bout activity levels compared to control flies. This confirms that the observed sleep phenotype in animals lacking *nowl* is not the result of hyperactivity but rather is a specific sleep-disruption phenotype.

**Fig 3:**
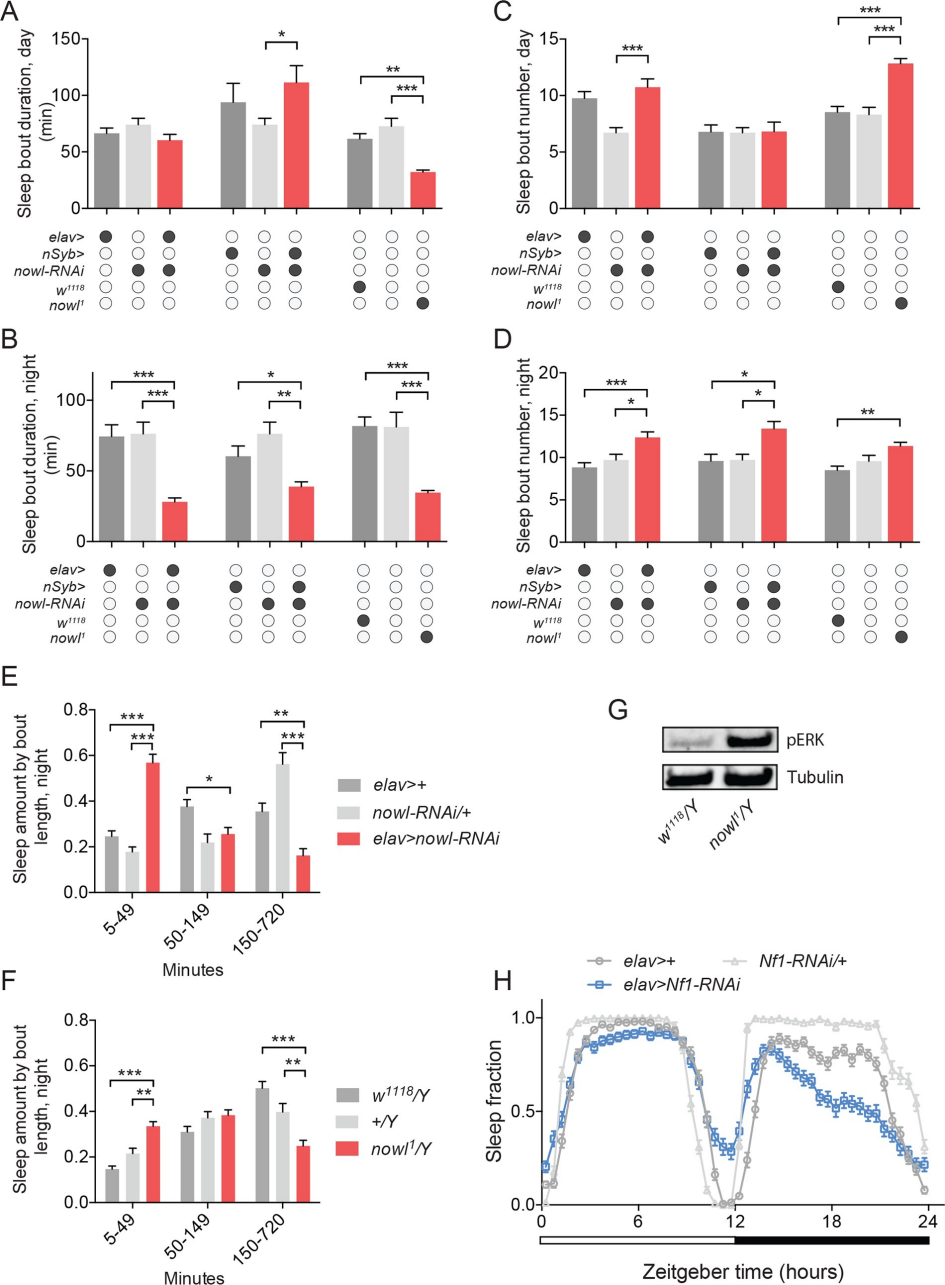
Loss of *nowl* in the nervous system causes sleep fragmentation. (A-D) Quantification of average sleep-bout duration (A and B) and number (C and D) during day and night for males with pan-neuronal knockdown of *nowl* using the stronger (*elav>*) and weaker (*nSyb>*) GAL4 drivers and the *nowl*^*1*^ insertional mutant. Neuronal knockdown of *nowl* using *elav>* (*elav>nowl-RNA*) and *nSyb>* (*nSyb>nowl-RNAi*) or mutation disrupting *nowl* (*nowl*^*1*^*/Y*) result in decreased sleep-bout length and increased sleep-bout number, with a stronger effect during night-time, compared to controls (*elav>+*, *nowl-RNAi/+*, *nSyb>/+*, *w*^*1118*^*/Y*, and *+/Y*, which does not carry any transgene or the *w*^*1118*^ mutation and therefore is denoted by open circles). (E-F) Fraction of time male animals spent sleeping in short (5-49-min.), medium (50-149-min.) or long (150-720-min.) sleep bouts during night for controls (*elav>+* and *nowl-RNAi/+*) compared to *elav>nowl-RNAi* animals and for *nowl*^*1*^ (*nowl*^*1*^*/Y*) mutants compared to controls (*w*^*1118*^*/Y* and *+/Y*). Flies carrying a *nowl* mutation or in which *nowl* has been knocked down neuronally spend significantly less time in long consolidated sleep episodes, compared to controls. (G) Western-blot analysis shows increased levels of phospho-ERK (pERK) in *nowl* mutant males compared to controls (*w*^*1118*^*/Y*), standardized to alpha-Tubulin. (H) Sleep pattern of *Neurofibromin-1* (*Nf1*) knockdown males under a 12-hour light/dark (white and black bars) cycle in 30-minute intervals shows effects on sleep compared to controls (*elav>+* and *Nf1-RNAi/+*) similar to animals lacking *nowl*, with decreased sleep mainly during the night. Graphs represent means with SEM (n = 32–92) of data pooled from one to three independent experiments. Significance was determined using Kruskal-Wallis test with Dunn's post-hoc testing (* p<0.05, ** p<0.01, *** p<0.001).

We next examined sleep fragmentation, which is associated with many psychiatric disorders and characterized by multiple short periods of sleep, *i*.*e*., an increased number of shorter sleep bouts. In the initial screen of genes within the 22q11.2 deletion, we found that knockdown of some genes including *slgA (PRODH)* and *nowl/LZTR1* also altered the number and duration of sleep bouts (S4A–S4D Fig). To further investigate whether loss of *nowl* plays a role in sleep maintenance following sleep initiation, we analyzed average sleep-bout number and length during day and night in males lacking *nowl*. Knockdown of *nowl* in the nervous system using either of the two pan-neuronal drivers *elav>* and *nSyb*-*GAL4* (*nSyb>*) caused a strong increase in fragmentation of night-time sleep, with a large decrease in sleep-bout duration combined with an increase in the number of sleep bouts, that was not observed during daytime (Fig 3A–3D and S5A–S5C Fig). Mutant flies carrying the *nowl*^*1*^ loss-of-function allele or the CRISPR-induced *nowl*^*KO*^ deletion exhibited similar night-time sleep-fragmentation patterns (Fig 3A–3D and S2E–S2H Fig).

To more closely examine the sleep changes associated with night-time sleep fragmentation, we analyzed the distribution of sleep episodes by binning them by duration and calculating the time spent in each bin, for flies with reduced *nowl* expression in the nervous system and for *nowl* mutants, compared to control genotypes. Neuronal knockdown of *nowl* significantly increased the amount of time spent in short sleep bouts of 5–49 minutes, compared to control flies, which consolidated most of their night-time sleep into longer bouts of 150–720 minutes (Fig 3E). A similar change was observed in *nowl* mutant flies (Fig 3F). These data indicate that *nowl* is required for maintenance of proper sleep architecture in *Drosophila*.

Human *LZTR1* was recently shown to function through the Cul3-based ubiquitin ligase complex, as a component of which it is suggested to mediate substrate specificity [28, 29]. Interestingly, *Cul3* has also been implicated as a causative gene in psychiatric disorders [30] and has been shown to play an important role in *Drosophila* sleep [15, 16, 31]. We wondered whether *nowl*, like its ortholog *LZTR1*, might exert its effects on sleep through an interaction with *Cul3*. We therefore knocked down *Cul3* pan-neuronally and analyzed sleep patterns, and found that *Cul3* silencing in the nervous system caused a significant decrease in average sleep-bout duration and a significant increase in average sleep-bout number (S6A–S6E Fig), during both day and night. This phenotype is similar to the sleep disruption caused by neuronal knockdown of *nowl*, which is consistent with an interaction between the two genes.

Mutations in human *LZTR1* have been linked with schwannomatosis, a form of neurofibromatosis, a neurological disorder associated with sleep problems, mental disabilities, and psychiatric disorders that can also be caused by mutation of the *Nf1* gene [18]. *Nf1* encodes a GTPase-activating protein that negatively regulates the Ras signaling pathway. We therefore asked whether *nowl*, like *Nf1*, affects Ras signaling, by measuring the levels of phosphorylated ERK (pERK), a downstream effector of Ras and an indicator of Ras-pathway activation. We found increased levels of pERK in heads from *nowl* mutants (Fig 3G), indicating that the Ras pathway is overactivated, consistent with previous findings [29]. Thus, the Nowl protein does appear to be required for normal Ras-pathway inhibition, either directly, like Nf1, as a negative regulator of Ras, or indirectly. We next investigated whether *Nf1* regulates sleep and found that neuronal knockdown of *Nf1* reduces night-time sleep nearly identically with loss of *nowl* (Fig 3H). These similarities are consistent with a sleep-regulating genetic interaction between *nowl* and *Nf1*. To exclude the possibility that the effect of *nowl* and *Nf1* on sleep is associated with increased neuronal proliferation or tumor formation, we next tested whether loss of these genes causes cell proliferation in the nervous system. Staining for phospho-Histone H3, a marker of mitosis, showed an absence of proliferating cells in the central nervous system of animals with knockdown of *nowl* or *Nf1* (S7A Fig). In humans, neurofibromatosis often leads to tumors in peripheral nerves, so we also tested whether loss of *nowl* or *Nf1* affects growth of peripheral nerves in *Drosophila* to exclude tumor formation in these neurons. Knockdown of *nowl* or *Nf1* in peripheral class-IV dendritic arborization (da) neurons did not lead to morphological abnormalities in these neurons, including their dendritic and axonal structures (S7B Fig). Together, this shows that knockdown of *nowl* or *Nf1* does not cause increased proliferation in the nervous system, suggesting that the associated phenotypes are caused by specific requirements for these genes in sleep-regulatory pathways.

**Fig 4:**
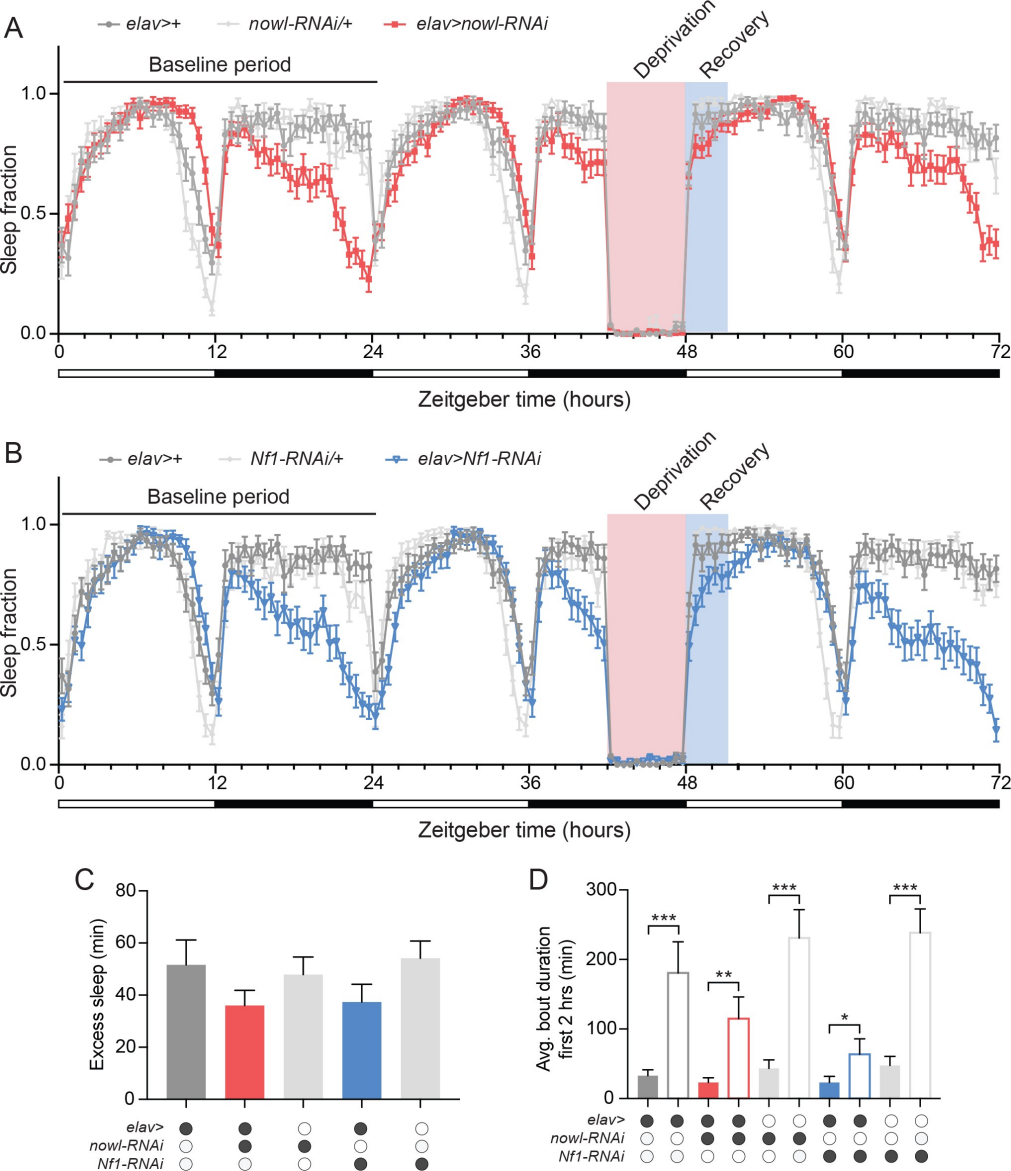
Homeostatic sleep recovery is not affected by neuronal loss of *nowl* or *Nf1*. (A and B) Daily sleep profiles of males under a 12-hour light/dark (white and black bars) cycle in 30-minute intervals for 72 hours for the first (baseline), second (deprivation–flies were subjected to mechanical sleep deprivation for the final 6 hours during night), and third (recovery) days in controls (*elav>+*, *nowl-RNAi/+*, and *Nf1-RNAi/+*) and animals with neuronal knockdown of *nowl* or *Nf1* (*elav>nowl-RNAi* and *elav>Nf1-RNAi*). Pink indicates sleep deprivation, while blue indicates recovery periods. (C) Quantification of recovery sleep (excess sleep) during the first 3 hours following sleep deprivation compared to the same period the first baseline day, shows rebound sleep for controls and animals with neuronal knockdown of *nowl* and *Nf1*. No significant differences were observed between genotypes. (D) Average duration of sleep-bouts initiated within the first 2 hours of the light phase was increased on the recovery day immediately following sleep deprivation compared to the first baseline day to a greater extent in controls than in animals with neuronal knockdown of *nowl* or *Nf1*. Filled bars represent the baseline day, and hollow bars represent the recovery day. Graphs represent means with SEM (n = 23–32) of data from one experiment. Significance was determined using Kruskal-Wallis test with Dunn's post-hoc testing (*p < 0.05, ** p < 0.01, *** p < 0.001).

In addition to circadian regulation, sleep is under homeostatic control, which allows recovery of lost sleep after insufficient sleep [32]. To analyze whether *nowl* or *Nf1* influences homeostatic sleep drive, we sleep-deprived animals and measured recovery sleep. To assess recovery sleep, we mechanically sleep-deprived flies for the final 6 hours during the night of the second day and measured sleep rebound. This treatment resulted in sleep loss in both controls and animals with neuronal knockdown of *nowl* or *Nf1* (Fig 4A and 4B). Following sleep deprivation, animals with neuronal knockdown of *nowl* or *Nf1* exhibited rebound sleep and recovered similarly to controls, showing the ability to recover lost sleep (Fig 4C). These results suggest that homeostatic recovery sleep is not altered by neuronal loss of *nowl* or *Nf1*. Animals increased their average sleep-bout length during the first two hours following sleep deprivation (Fig 4D). However, this was less pronounced in flies with neuronal knockdown of *nowl* or *Nf1*, indicating impaired sleep consolidation with increased fragmentation.

### Interactions between *nowl* and *Nf1* suggest that they act on a common sleep-regulatory pathway

**Fig 5:**
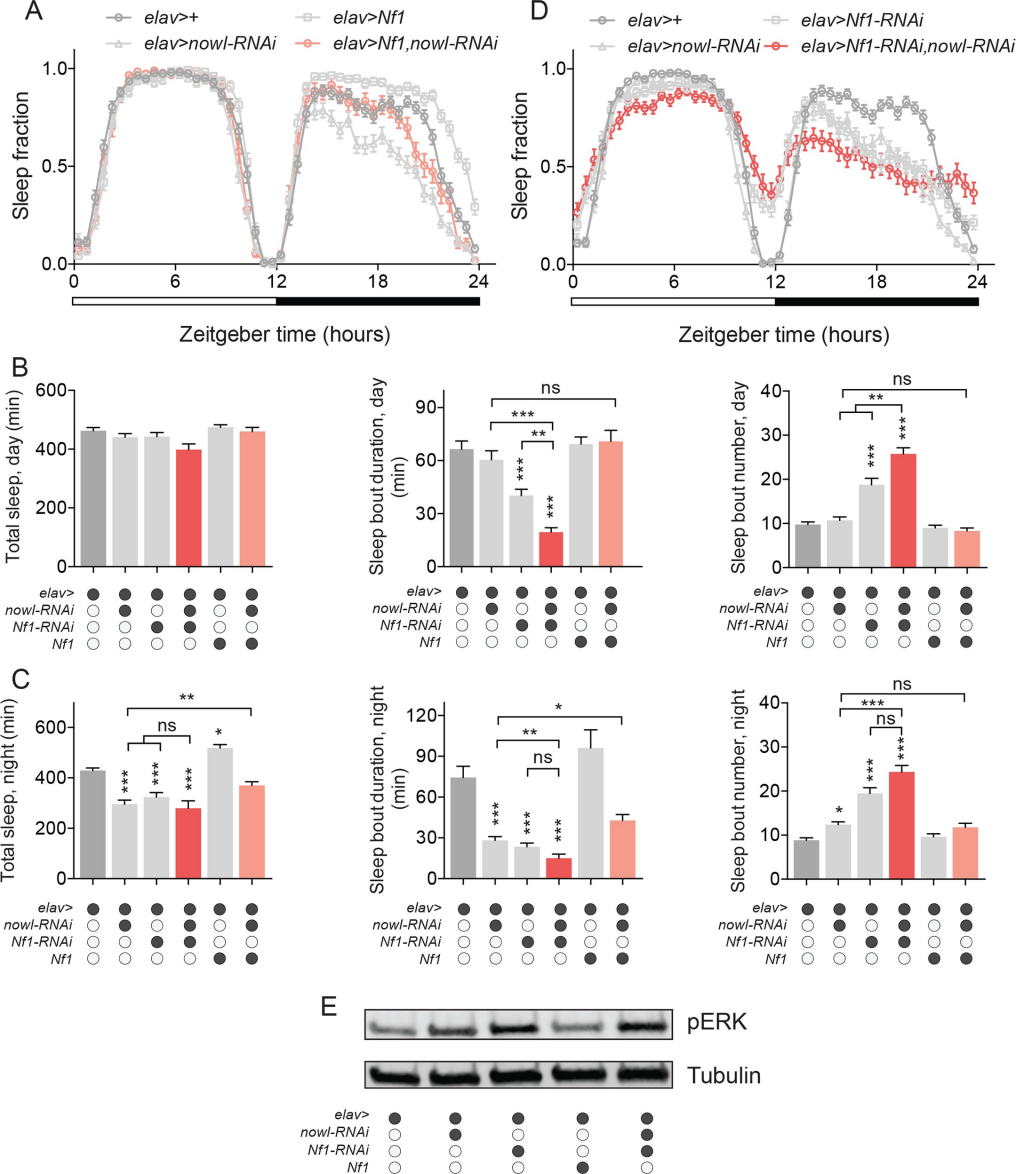
*nowl* and *Nf1* interact in the regulation of sleep. (A) Daily sleep profiles of males under a 12-hour light/dark (white and black bars) cycle in 30-minute intervals. In *elav>Nf1*, *nowl-RNAi* animals (with neuronal overexpression of *Nf1* and simultaneous knockdown of *nowl*), night-time sleep is partially restored compared to *nowl* knockdown alone. (B and C) Quantification of daytime (B) and nighttime (C) total sleep, sleep-bout duration, and sleep-bout numbers in males. The double knockdown (*elav>nowl-RNAi*, *Nf1-RNAi*) shows significant interactions between *nowl* and *Nf1* in daytime total sleep and sleep-bout duration and number, indicating an additive effect in the daytime. The effects of double knockdown on total nighttime sleep and nighttime sleep-bout duration and number are not different from *Nf1-RNAi* alone, suggesting an epistatic relationship between *nowl* and *Nf1* in night-time sleep regulation. *Nf1* overexpression partially rescues the *nowl-*knockdown effect on sleep. Controls are *elav>+*. Additional effector line controls (*nowl-RNAi/+*, *Nf1-RNAi/+*, and *Nf1/+*) are shown in S8 Fig Genotypes that are indicated as different from the *elav>+* controls are also statistically different (P< 0.05) from controls shown in S8 Fig (D) Daily sleep profiles of double-knockdown male animals (*elav>Nf1-RNAi*, *nowl-RNAi*) display a pronounced decrease in sleep. Controls are *elav>+*. (E) Western blotting against phosphorylated ERK (pERK) in males shows that single and double knockdowns of *nowl* and *Nf1* cause increased pERK levels, standardized to alpha-Tubulin (controls are *elav>+*). Western blot is representative of two independent experiments. Graphs represent means with SEM (n = 32–81) of data pooled from one to three independent experiments. Significance was determined using Kruskal-Wallis test with Dunn's post-hoc testing (*p < 0.05, ** p < 0.01, *** p < 0.001).

To investigate the possibility of genetic interaction between *nowl* and *Nf1* in the regulation of sleep, we overexpressed *Nf1* in *nowl-RNAi* animals. In this test, a rescue of the *nowl* short-sleep phenotype would indicate that *Nf1* functions downstream of *nowl* in a sleep-regulatory pathway. Neuronal overexpression of *Nf1* partially rescued the loss of night-time sleep and sleep fragmentation caused by *nowl* knockdown (Fig 5A–5C and S8 Fig), suggesting that *nowl* and *Nf1* may function in the same sleep-regulatory pathway, with Nf1 working downstream of Nowl. Next, we analyzed interaction by comparing effects of the single-hit knockdown compared to the double-hit knockdown of *nowl* and *Nf1*. As an initial step, we determined the efficiency and specificity of the RNAi-mediated knockdown using qPCR, and validated that the RNAi lines against *nowl* and *Nf1* both significantly reduced target-gene expression (S9A and S9B Fig). In animals with simultaneous knockdown of *nowl* and *Nf1* in the nervous system, sleep fragmentation was greater than in animals with knockdown of either *nowl* or *Nf1* alone and increased compared to the control, indicating that *nowl* and *Nf1* interact in the regulation of daytime sleep (Fig 5B and 5D). In animals with double knockdown of *nowl* and *Nf1*, total night-time sleep was reduced, similar to animals with single knockdown of *nowl* or *Nf1*, compared to the control (Fig 5C). Simultaneous knockdown of *nowl* and *Nf1* also increased night-time sleep fragmentation compared to the control. However, the effect of the double knockdown on night-time sleep parameters largely resembled the effect of *Nf1* knockdown alone, suggesting an interaction between *nowl* and *Nf1* in night-time sleep regulation that may be epistatic. The ability of *Nf1* overexpression to partially rescue the overall loss of night-time sleep and the reduced sleep-bout duration during the night supports the notion that these two genes are functionally related and may converge on a common sleep-regulatory pathway. To exclude indirect developmental effects, we next asked whether *nowl* and *Nf1* influence sleep through effects occurring during development or whether these genes have acute effects on sleep in the adult brain. Using the RU486-activated GeneSwitch system [33] to restrict knockdown to the adult stage and thus to avoid indirect developmental effects, we induced neuronal knockdown of *nowl* and *Nf1* in adults. Adult-specific neuronal knockdown of *nowl* or *Nf1* decreased night-time sleep compared to controls (S10A and S10B Fig) and caused an increased number of short sleep bouts during the night (S10C–S10F Fig). These adult-specific manipulations suggest that the sleep phenotypes caused by loss of *nowl* and *Nf1* are unlikely due to developmental effects and that adult-specific function of Nowl and Nf1 is important for normal sleep.

**Fig 6:**
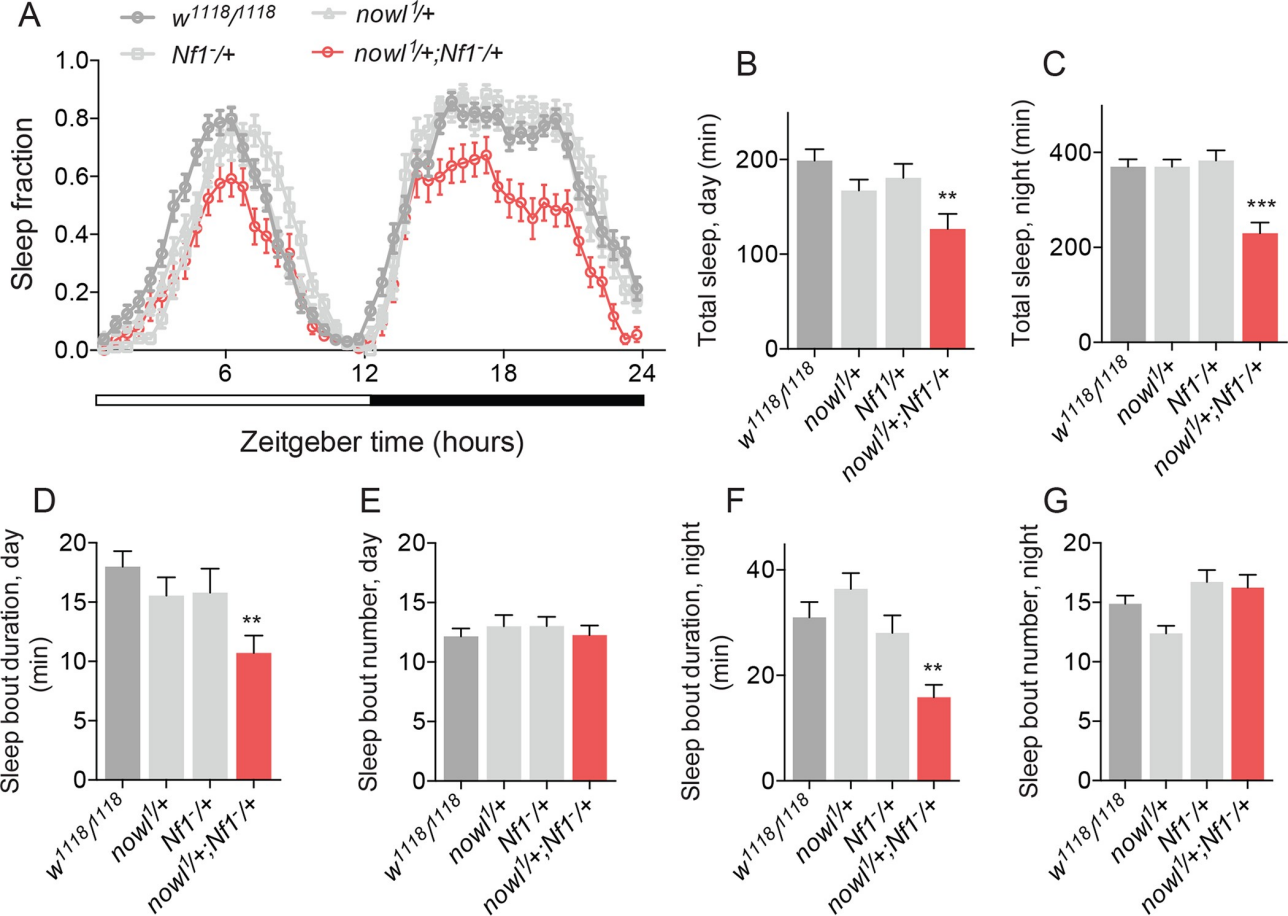
Interactions between *nowl* and *Nf1* suggest that they act on a common sleep-regulatory mechanism. (A) Sleep patterns of heterozygous *nowl*^*1*^*/+* and *Nf1*^*-*^*/+* females show that one copy of each is sufficient for maintaining proper sleep, while the trans-heterozygous (*nowl*^*1*^*/+*; *Nf1*^*-*^*/+*) mutants exhibit decreased sleep during both day and night. (B-G) Quantification of sleep parameters shows reduced average sleep-bout duration of trans-heterozygous *nowl*^*1*^*/+; Nf1*^*-*^*/+* females, indicating that *nowl* and *Nf1* interact to maintain sleep. Graphs represent means with SEM (n = 32) of data from one experiment. Significance was determined using Kruskal-Wallis test with Dunn's post-hoc testing (*p < 0.05, ** p < 0.01, *** p < 0.001).

Since our results indicated that Nowl inhibits the Ras pathway (Fig 3G), we investigated the combined effect of *nowl* and *Nf1* loss on the activation of the Ras pathway. Our data show that silencing *nowl* or *Nf1* individually in the nervous system results in Ras overactivation, reflected in increased pERK levels (Fig 5E). Consistent with this and with their genetic interaction in sleep regulation, the simultaneous knockdown of *nowl* and *Nf1* activates the Ras pathway to an extent similar to knockdown of *Nf1* alone. Together these effects indicate that *nowl* and *Nf1* interact to suppress Ras-pathway activity. To investigate the genetic interaction between *nowl* and *Nf1* further, we compared the phenotypes of *Nf1* and *nowl* trans-heterozygous mutants with those of the individual heterozygous mutants. The *nowl* gene is on the X chromosome, and males are therefore hemizygous; heterozygous effects can only be studied in females. Female animals heterozygous for either *nowl* or *Nf1* displayed similar sleep patterns, with no significant loss of night-time sleep compared to the control (Fig 6A–6C), whereas trans-heterozygous *nowl*^*+/-*^ *Nf1*^*+/-*^ mutant females exhibited a strong reduction in total sleep. Furthermore, trans-heterozygous *nowl*^*+/-*^ *Nf1*^*+/-*^ mutants displayed shorter sleep bouts, while animals heterozygous for mutations in either *nowl* or *Nf1* alone displayed no change in sleep-bout duration compared to controls (Fig 6D–6G). This indicates that trans-heterozygous loss of both *nowl* and *Nf1* causes sleep fragmentation, further supporting the existence of a genetic interaction between *nowl* and *Nf1* and a functional relationship between these two genes in the regulation of sleep.

### Neuronal *Nf1* and *nowl* are required to maintain metabolic homeostasis

**Fig 7:**
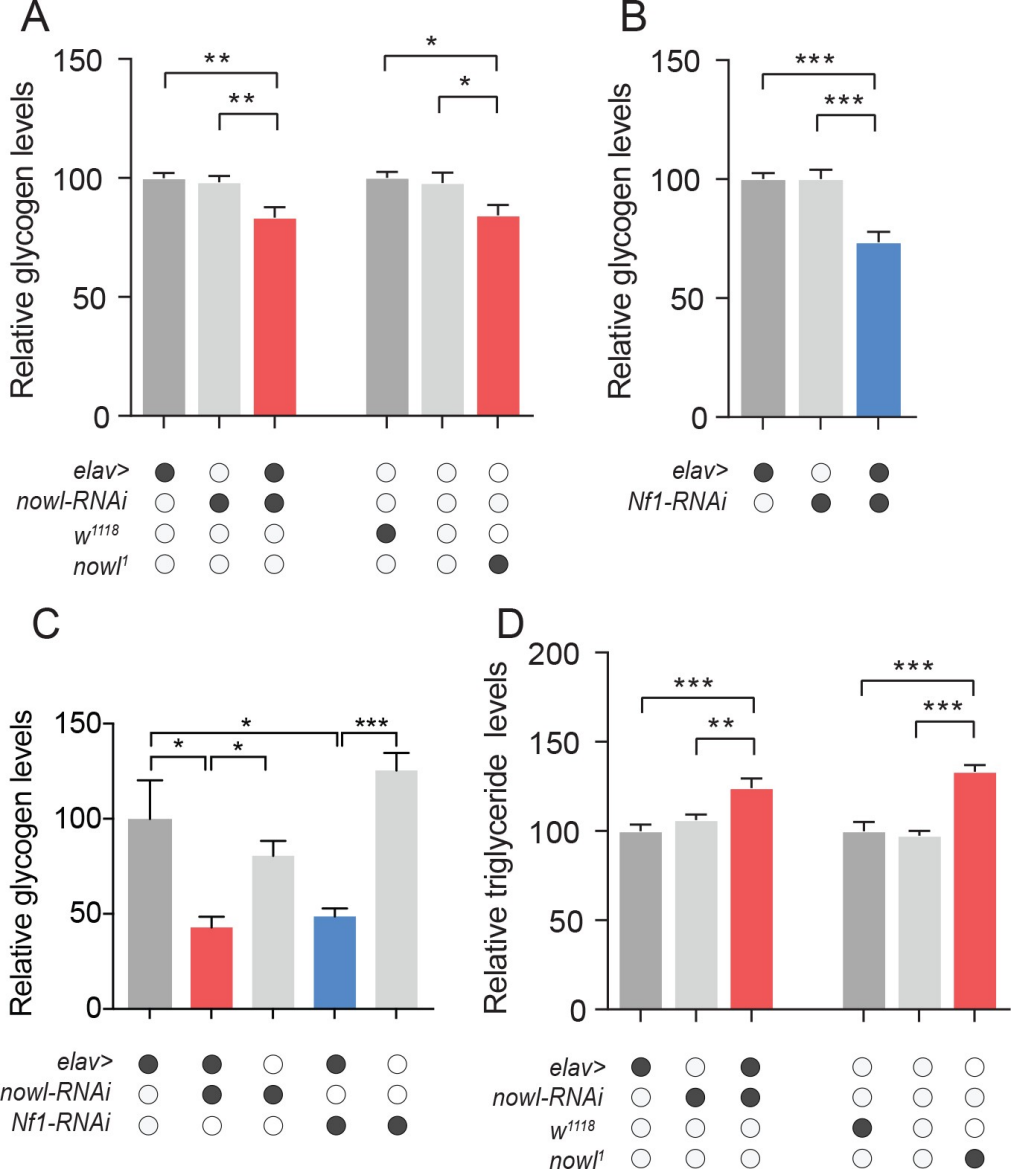
Loss of *nowl* and *Nf1* leads to physiological changes that affect energy homeostasis. (A-C) Quantification of whole-body glycogen (A, B) and triglyceride (D) content, and head glycogen (C), in adult male flies. Neuronal knockdown of *nowl* (*elav>nowl-RNAi*) or *Nf1* (*elav>Nf1-RNAi*) or mutation disrupting *nowl* (*nowl*^*1*^*/Y*) results in reduced whole-body glycogen levels (A, B) compared to controls (*elav>+*, *nowl-RNAi/+*, *Nf1-RNAi/+*, *w*^*1118*^*/Y*, and *+/Y*). (C) Effect of neuronal *nowl* and *Nf1* knockdown on glycogen content of the head. (D) Whole-body lipid content is increased in animals with neuronal knockdown of *nowl* or a mutation disrupting *nowl*. Graphs represent means with SEM (n = 5–20) of data from one experiment. Significance was determined using Kruskal-Wallis test with Dunn's post-hoc testing (*p < 0.05, ** p < 0.01, *** p < 0.001).

Emerging evidence suggests that sleep is important for energy homeostasis and that insufficient sleep can lead to obesity [34]. A proposed main function of sleep is to provide an opportunity to replenish brain supplies of glycogen, which are depleted during periods of wakefulness [35, 36]. Since *nowl* and *Nf1* loss decrease night-time sleep and thereby increase wakefulness, we analyzed glycogen levels in these animals. Consistent with the notion that sleep is important for maintaining glycogen levels, we found that indeed whole-body glycogen levels were reduced in animals with neuronal knockdown of *nowl* and *Nf1* and in *nowl* mutants (Fig 7A and 7B), which exhibits reduced night-time sleep. These data further suggest that neuronal function of *nowl* and *Nf1* is essential for maintenance of glycogen stores, which may be related to their importance for getting enough sleep. Since sleep deprivation reduces brain glycogen [37], we asked whether neuronal knockdown of *nowl* or *Nf1*, which causes reduced night-time sleep, affects the glycogen content of the head. Heads of animals with neuronal knockdown of *nowl* or *Nf1* showed reduced glycogen levels (Fig 7C), suggesting that *nowl* and *Nf1* play a role in the regulation of brain glycogen metabolism. We next analyzed whole-body triglyceride levels, since the 22q11.2 deletion is associated with increased risk of obesity, which is also linked to insomnia [38, 39]. We found increased organismal levels of triglycerides in flies with neuronal knockdown of *nowl* and in *nowl* mutants (Fig 7D), suggesting that they exhibit increased adiposity, like human 22q11.2 carriers. Taken together our results suggest that *nowl* is required in the nervous system to maintain organismal energy homeostasis, a function that might be related to its role in sleep, considering the vital role of sleep in the regulation of energy homeostasis.

### *Nf1* and *nowl* are required in GABA-responsive cells for night-time sleep

**Fig 8:**
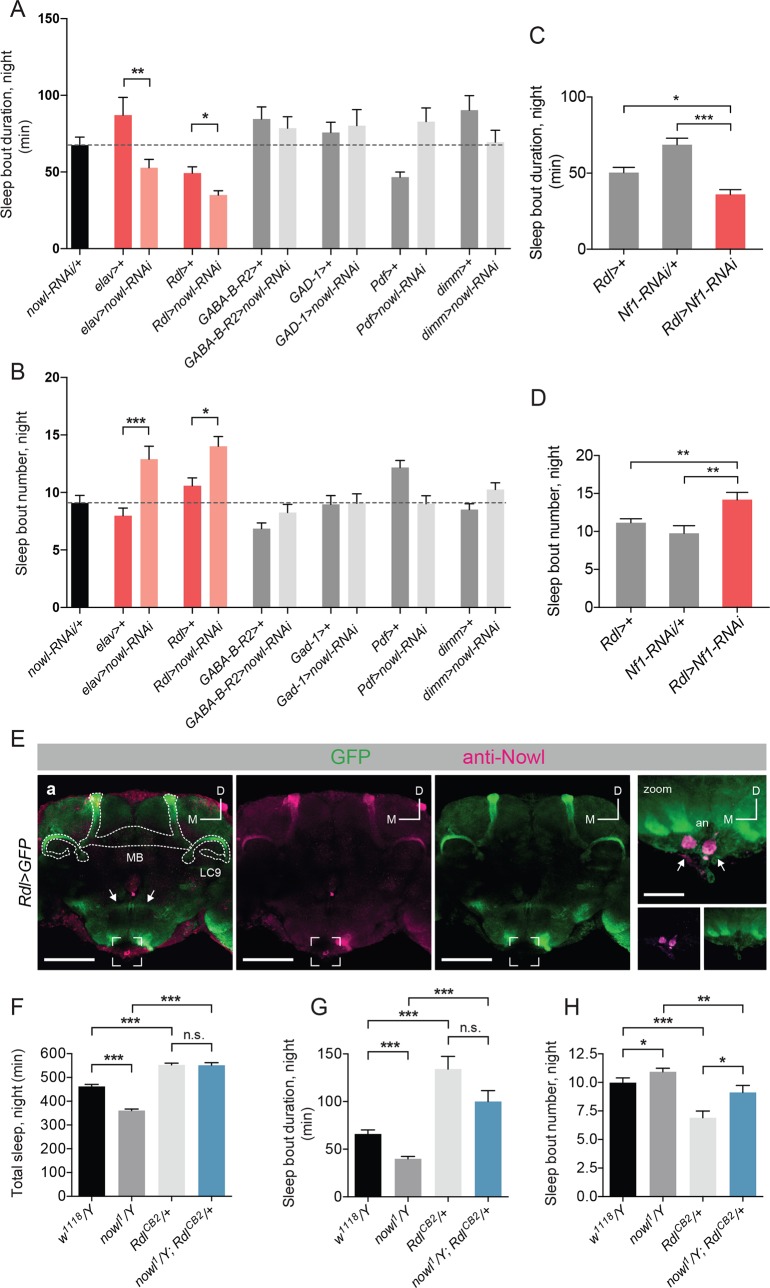
*nowl* is required in GABA-responsive neurons for sleep maintenance, and increased GABA signaling rescues the sleep phenotype of *nowl* mutants. (A-D) Quantification of average male night-time sleep-bout duration and length. (A) Sleep-bout duration is decreased, and (B) sleep-bout number is increased during the night when *nowl* is knocked down pan-neuronally (*elav>nowl-RNAi*) or specifically in GABA_A_-receptor *Rdl*-expressing neurons (*Rdl>nowl-RNAi*) compared to controls (*elav>*+ and *Rdl>+*), respectively. Knockdown animals were compared to the *GAL4* driver line crossed to *w*^*1118*^ (the genetic background for the RNAi), and *elav>nowl-RNAi* was used as a positive control. (C) Sleep-bout duration is decreased and (D) sleep-bout number increased in animals with knockdown of *Nf1* in *Rdl*-expressing neurons compared to controls (*Rdl>+* and *Nf1-RNAi/+*). (E) Anti-Nowl staining (purple) in males is prominent in *Rdl*-expressing neurons (*Rdl>GFP*, green) of the mushroom body (MB), among other sites. The neurons of the α′/β′ lobes of the MB (outlined; “MB”) and the neurons projecting to the LC9 optic glomerulus (outlined; “LC9”) express both *Rdl>GFP* and Nowl. Interneurons connecting the paired olfactory lobes (arrows) express *Rdl>GFP* but not Nowl, and a pair of neuronal clusters at the tip of the subesophageal ganglion (bracketed and zoomed in right panel) strongly express Nowl but not *Rdl>GFP*, indicating independent expression and staining. (F-H) Quantification of male night-time total sleep and average sleep-bout duration and number for *w*^*1118*^*/Y*, *nowl*^*1*^*/Y*, *Rdl*^*CB2*^*/+*, and *nowl*^*1*^*/Y; Rdl*^*CB2*^*/+*. (F) The reduced night-time sleep exhibited by *nowl*^*1*^ mutant flies is rescued by introducing one copy of the *Rdl*^*CB2*^ allele. (G, H) Sleep-bout duration during the night is significantly decreased in *nowl* mutants, and sleep-bout number is significantly increased. Introducing one copy of the *Rdl*^*CB2*^ allele partially rescues both defects. Graphs represent means with SEM (n = 32–156) of data pooled from one to five independent experiments. Significance was determined using Kruskal-Wallis test with Dunn's post-hoc testing (* p<0.05, ** p<0.01, *** p<0.001).

Changes in neuronal circuitry and neurotransmitter systems have been proposed to underlie several neuropsychiatric disorders. Signaling through GABA, in particular, has been found to be an important contributor to sleep in mammals [40]. GABAergic signaling has likewise been found to play a role in *Drosophila* night-time sleep initiation and maintenance [41–43]. Because we observed a strong disruption of both of these parameters in *nowl* knockdown and mutant flies, we wondered whether this gene might have a function in GABAergic signaling. To investigate this, we knocked down *nowl* in GABA-producing or GABA-responsive neurons using a panel of *GAL4* driver lines. GABA-producing neurons were targeted using *Gad1-GAL4* (*Gad1>*), which drives expression in neurons expressing the GABA-biosynthetic enzyme glutamate decarboxylase 1 (GAD 1). *Rdl-GAL4* (*Rdl>*) was used to drive expression in neurons expressing the ionotropic GABA_A_ receptor, while *GABA-B-R2-GAL4* (*GABA-B-R2>*) was used to target neurons expressing the metabotropic GABA_B_ receptor subtype 2. Furthermore, GABAergic innervation has been shown to regulate “clock” neurons expressing the Pigment Dispersing Factor (PDF) peptide, comprising a limited number of wake-promoting neurons, the small and large ventral lateral neurons (s-LN_v_ and l-LN_v_, respectively) [32, 44]. To assess the function of *nowl* in these neurons, the *Pdf-GAL4* (*Pdf>*) driver was used to drive expression in all PDF-expressing neurons, while the *dimm-GAL4* (*dimm>*) driver allowed for knockdown in a broad array of peptidergic neurosecretory cells, including the l-LN_v_s, suggested to be the only sleep-regulatory neurons in this subset of cells [45]. Since global or neuronal loss of *nowl* leads to highly fragmented night-time sleep, we asked whether knockdown of *nowl* in any of these restricted neuronal subsets could elicit a similar effect on sleep maintenance. Interestingly, we found that knockdown of *nowl* using the *Rdl>* driver, thus specifically in GABA_A_-receptor-expressing neurons, caused a shortening of sleep bouts and an increase in their number during night, with an effect size similar to that seen with pan-neuronal knockdown (Fig 8A and 8B). *Rdl* encodes the *Drosophila* GABA_A_ receptor, a ligand-gated chloride channel that mediates the fast inhibitory effects of GABA, and it has been shown to affect several behaviors such as learning and sleep [43, 46]. Knockdown of *nowl* in other neuronal populations using the other drivers–targeting the GABA-producing cells, the GABA_B_-receptor-expressing neurons, and the PDF-expressing clock neurons–had no significant effect on night-time sleep-bout number or duration.

Since our data show that *nowl* interacts genetically with *Nf1* in the regulation of night-time sleep (Figs 5 and 6), we asked whether *Nf1*, like *nowl*, also regulates sleep maintenance through specific effects in the GABA_A_-receptor Rdl-expressing neurons. Consistent with the interaction of *nowl* and *Nf1* in sleep regulation, knockdown of *Nf1* in *Rdl*-expressing neurons produced a sleep-fragmentation phenotype characterized by more frequent sleep bouts of decreased duration, similar to loss of *nowl* in these GABA-responsive neurons (Fig 8C and 8D). Together these data suggest that both *nowl* and *Nf1* are specifically required in GABA-responsive *Rdl*-expressing neurons to maintain sleep after sleep onset. The lack of knockdown effect in the PDF-expressing neurons further supports the independence of the night-time sleep phenotype of *nowl*-knockdown flies from circadian clock function.

To investigate the expression of Nowl in the brain, particularly in GABA_A_-receptor *Rdl-*expressing neurons, we generated an anti-Nowl antibody. Colocalization of mCD8::GFP expression in the *Rdl*-positive neurons with staining against Nowl shows that the mushroom bodies (MBs) express both the GABA_A_-receptor *Rdl* and Nowl (Fig 8E). Interestingly, downregulation of *Rdl* in the α′/β′ neurons of the MBs causes sleep loss similar to knockdown of *nowl* [47]. Together these data are consistent with a model in which Nowl promotes night-time sleep via modulation of GABA signaling that regulates the activity of the Rdl-expressing mushroom-body α′/β′ lobes.

### Increased inhibitory signaling through the GABA_A_ receptor rescues *nowl* mutant sleep phenotype

Knockdown of *nowl* in GABA_A_-receptor *Rdl*-expressing neurons led to disruptions in sleep architecture, similar to the effects observed when decreasing inhibitory GABAergic signaling via Rdl [47]. We therefore hypothesized that loss of *nowl* might affect sleep by reducing inhibitory GABAergic signaling effects in these neurons, thus disinhibiting them, which should promote wakefulness and reduce sleep [42, 43]. To address this possibility, we asked whether the *Rdl*^*CB2*^ mutation would rescue the *nowl* mutant sleep phenotype. *Rdl*^*CB2*^ mutant flies express a form of the GABA_A_ receptor Rdl that is thought to exhibit reduced desensitization, leading to longer channel-opening duration, increased channel flux, and therefore increased neuronal inhibition downstream of GABA reception [41]. We rationalized that if loss of *nowl* increases neuronal excitability due to loss of GABA_A_-receptor signaling in *Rdl*-expressing neurons, the mutant Rdl^CB2^ receptor might compensate for this and thereby rescue the sleep disturbance observed in *nowl* loss-of-function flies. Compared to control flies, introduction of one copy of the *Rdl*^*CB2*^ allele significantly increased total sleep during the night, consistent with the expected effect of increased inhibitory GABA signaling. As we previously observed, *nowl* mutant flies displayed significantly decreased total sleep during the night. This effect was completely rescued in flies carrying both *nowl*^*1*^ and *Rdl*^*CB2*^ (Fig 8F and S11 Fig). The addition of *Rdl*^*CB2*^ caused a decreased number of longer night-time sleep bouts, eliminating the night-time sleep fragmentation induced by the loss of *nowl* function (Fig 8G and 8H). Taken together, these data indicate that increased inhibitory signaling through the GABA_A_ receptor Rdl largely rescues the sleep disturbances observed in *nowl* loss-of-function flies. We thus propose that *nowl* affects sleep by modulating GABA signaling in *Rdl*-expressing neurons.

## Discussion

Schizophrenia is a highly heritable disorder with a prevalence of 1% in the population [48, 49]. It encompasses a wide spectrum of symptoms, of which a major one is sleep disturbance [9]. The human 22q11.2 chromosomal deletion spans more than 40 genes, and children carrying this deletion are 20 to 25 times more prone to developing schizophrenia during adolescence than their peers [50]. To identify which 22q11.2 genes might underlie the syndrome and development of schizophrenia in human carriers, we screened for conserved genes within this deletion that are required for sleep in *Drosophila* by RNAi-induced knockdown in the nervous system. Neuronal knockdown of several genes each caused abnormal sleep patterns and activity in flies. In females, silencing of several genes including *CDC45L* (human *CDC45*), *CG13192* (*GNB1L*), *Es2* (*DGCR14*), and *Septin4* (*SEPT5*) resulted in short-sleep phenotypes. Some of these genes have not previously been linked to behavioral deficits in schizophrenia. However, we observed that neuronal knockdown of *CG13192* (human *GNB1L*) and *slgA* (*PRODH*) resulted in an increased number of sleep episodes that have a shorter length. PRODH catalyzes a step in the conversion of proline to glutamate, while *GNB1L* encodes a G-protein beta-subunit-like polypeptide [51, 52]; both genes have also been associated with schizophrenia and other mental disorders [51, 53–55]. In males, neuronal knockdown of several genes increased total sleep, while only *nowl* knockdown caused a decrease in total sleep.

We show here that *nowl*, the conserved *Drosophila* homolog of the human *LZTR1* gene located within the 22q11.2 deletion, is required for night-time sleep initiation and maintenance. Mutation or neuronal knockdown of *nowl* decreased total sleep amount and caused highly fragmented sleep, especially during night-time, which is a phenotype frequently seen in mental illness [27]. Schizophrenia is a neurodevelopmental disorder believed to involve developmental pathology [56]. Our findings show that knockdown of *nowl* or *Nf1* both during development and only in the adult stage results in reduced and fragmented night-time sleep. Specific knockdown in the adult stage also results in similar sleep phenotypes, indicating that *nowl* and *Nf1* are required in the adult stage for normal night-time sleep. However, developmental effects cannot be completely excluded by these experiments with knockdown at the time of adult eclosion, since some changes within the nervous system occur post-eclosion.

In addition, *nowl* mutants exhibit highly delayed sleep onset (*i*.*e*., increased sleep-onset latency). Sleep-onset latency is a widely used measure in the study of human sleep disorders and psychiatric illnesses, including schizophrenia [27]. Accumulating evidence also indicates that sleep sufficiency is important for the maintenance of energy balance and that reduced or poor sleep increases the risk of developing obesity [34]. Sleep is tightly connected to metabolic processes, and one proposed function of sleep is that it serves a key role in replenishing glycogen stores, which are depleted during wakefulness [35, 36]. Consistent with this notion, brain glycogen levels are highest during periods of sleep and decrease following rest deprivation in *Drosophila* [37]. Our finding of reduced glycogen levels in whole body and heads of flies lacking *nowl* or *Nf1* function is in alignment with the view that sleep disruption is associated with depletion of glycogen stores, although further studies are needed to determine whether this effect is linked to alterations in sleep or to more-direct effects on metabolism. Furthermore, our results show increased adiposity associated with loss of *nowl* in flies, suggesting that *LZTR1* loss may contribute to the increased genetic susceptibility to develop obesity found in human 22q11.2 carriers [38]. Taken together the defects in sleep initiation and maintenance observed in *nowl* mutants suggest that *LZTR1* loss may contribute to the symptoms observed in the 22q11.2 DS and in schizophrenia, including the increased incidence of obesity.

Delayed sleep onset and the short-sleep phenotype could be caused either by alterations in sleep homeostasis or by disturbances in circadian rhythm. Our data indicate that animals lacking *nowl* function exhibit a normal circadian period length and suggests that their reduced night-time sleep is not related to problems with homeostatic sleep regulation. Animals with neuronal loss of *nowl* or *Nf1* have a normal capacity to recover lost sleep at beginning of the day, although this does not rule out night-time effects. Furthermore, the night-time short sleep of animals with loss of *nowl* function is not a consequence of hyperactivity, which demonstrates that the sleep fragmentation observed in these animals is a specific sleep-disturbance phenotype, likely associated with inability to sustain long periods of sleep. Since *nowl* encodes a highly conserved protein (51% identity and 67% similarity to the human LZTR1 protein), Nowl may govern an evolutionarily conserved mechanism that regulates night-time sleep in animals.

Human LZTR1 has been shown to interact with Cul3 in the Cul3 ubiquitin-ligase complex [28]. Cul3 in *Drosophila* has been associated with sleep and interacts physically in this regulation with Insomniac, another BTB/POZ-domain protein (like Nowl) that functions as a substrate adaptor in the Cul3 complex [16, 31]. Although *Cul3* knockdown and *nowl* knockdown are phenotypically similar, *Cul3* knockdown induces broader phenotypes including disruption of the circadian clock [15]. Future studies should determine whether Nowl regulates sleep through a physical interaction with Cul3. Recent studies have found that LZTR1 may function in the Cul3 ubiquitin ligase complex to ubiquitinate Ras [28, 29, 57], which has previously been implicated in sleep regulation [58]. Nf1 is another important regulator of Ras signaling, and mutations in *Nf1* and *LZTR1* both give rise to types of neurofibromatosis [18, 59]. We show that *nowl* is required for proper negative regulation of Ras signaling, similar to *Nf1*, and that *nowl* and *Nf1* interact in the regulation of sleep. Heterozygotes for either *nowl* or *Nf1* mutations exhibit normal sleep patterns, while *nowl Nf1* trans-heterozygous animals display disrupted sleep architecture, including sleep fragmentation, indicating a genetic interaction. Furthermore, the reduction in total sleep and the fragmentation of night-time sleep caused by loss of *nowl* are partially rescued by *Nf1* overexpression. Thus, these two genes may function in the same pathway, or both of them may act on a common element involved in sleep maintenance. Whether *nowl* and *Nf1* regulate sleep through their effects on Ras activity will be an interesting question for future studies.

GABA neurotransmission is often altered in schizophrenic patients, and it is the main system that regulates night-time sleep [41, 44, 60, 61]. Since disruption of GABA signaling has also been shown to reduce night-time sleep in *Drosophila*, we asked whether *nowl* and *Nf1* might function in GABA-producing or -responsive cells. We found that knockdown of *nowl* in *Rdl*-expressing GABA-responsive neurons led to strongly increased sleep fragmentation, similar to the effect of pan-neuronal *nowl* knockdown. The wake-promoting l-LN_v_ clock neurons express Rdl and play a major role in regulating sleep [43]. When these neurons are artificially hyper-excited by the expression of the sodium channel NaChBac, which allows membrane depolarization to occur more readily, flies showed decreased levels of sleep and increased levels of arousal, especially during night-time [45]. Knockdown of *Rdl* in these cells increases sleep-onset latency [42], while reduced *GABA*_*B*_*-R-2* metabotropic receptor expression leads to reduced sleep during the late night [44]. Using RNAi-induced knockdown driven by the *Pdf>* and *dimm>* constructs, we found that *nowl* does not appear to be required in these s-LN_v_ clock neurons to regulate sleep, indicating that other *Rdl*-expressing neurons are involved in sleep-regulatory *nowl* activity. The mushroom bodies (MBs), which are required for olfactory associative learning and memory [62] and which also regulate sleep [63, 64], strongly express Rdl (Fig 7E and [46]). A single pair of dorsal paired medial (DPM) neurons have been found to strongly promote sleep via GABAergic and serotonergic innervation of the mushroom bodies [47]. The MB α′/β′ neuronal population in particular are wake-promoting neurons that receive GABAergic input from the DPM neurons. Increased activation of the α′/β′ population decreases night-time sleep and increases sleep fragmentation [47]. Knockdown of *Rdl* in the α′/β′ neurons results in night-time sleep-loss phenotypes almost identical to those seen with knockdown of *nowl* either pan-neuronally or specifically in the *Rdl*-expressing neurons, inducing increased sleep-bout numbers and reduced bout lengths, with a stronger effect during night-time [47]. Furthermore, we found strong enrichment of Nowl in *Rdl*-expressing MB neurons, consistent with Nowl’s functioning in the regulation of sleep via wake-promoting neurons in the mushroom body. Interestingly, *nowl* was also identified in an olfactory-learning screen, in which it was found to inhibit electric-shock-reinforced associative learning, consistent with a role for *nowl* in mushroom-body neuronal function [26]. Furthermore, we observed that *Nf1* knockdown in *Rdl*-expressing neurons caused sleep reduction and fragmentation of night-time sleep similar to *nowl* knockdown, further supporting an interaction between *Nf1* and *nowl* in the regulation of sleep. Investigating whether *nowl* and *Nf1* do indeed regulate sleep via effects on GABA signaling in the mushroom bodies will be an interesting subject for follow-up studies.

It has been suggested that mutations in ion channels can lead to disrupted sleep by altering overall neuronal excitability [65]. One example of this is the gene *Shaker*, which encodes an α-subunit of a potassium channel that functions to regulate membrane repolarization after neuronal depolarization [66]. Loss-of-function mutations in this gene, and in its mouse orthologs, cause reduced sleep and shorter sleep episodes, without affecting circadian or homeostatic sleep drive [67]. Since sleep loss and fragmentation due to loss of *nowl* were partially rescued by increased GABAergic inhibition of *Rdl*-expressing neurons, we suggest that loss of *nowl* may modulate GABA signaling in wake-promoting *Rdl*-expressing neurons. It will be interesting to determine whether *nowl* directly affects the Rdl receptor or other substrates via Cul3 to regulate postsynaptic GABA signaling. Cul3 and its interaction partner Insomniac are recruited to the postsynaptic compartment within minutes of acute glutamate-receptor inhibition [68]. These proteins mediate local mono-ubiquitination, which is important for homeostatic signaling in the postsynaptic compartment. The number of Rdl receptors expressed in a neuron, beyond the mere presence or absence of these receptors, has been suggested to be important in sleep regulation [43]. Furthermore, in humans, agonists of GABA_A_-type receptors are common treatments for insomnia [69], and a loss-of-function mutation affecting a GABA_A_ receptor subunit causes a heritable type of insomnia [70]. Altered GABAergic signaling is believed to play a role in many neurodevelopmental disorders, and a mechanism that involves GABA_A_-receptor signaling may be a key factor underlying the pathophysiology of the 22q11.2 DS. Our findings suggest a contribution of *nowl/LZTR1* –a 22q11.2 gene–in altered GABAergic neurotransmission and thus may provide a new direction for understanding the mechanisms underlying the observed neurodevelopmental phenotypes in the 22q11.2 DS and for developing therapeutic interventions against them.

Essentially all psychiatric disorders are associated with sleep disturbances. We have identified *nowl* as regulator of sleep in *Drosophila*. Mutations in its human ortholog *LZTR1* have been implicated in several human diseases, such as schwannomatosis, glioblastoma, and the psychiatric disorders linked to the 22q11.2 DS. We suggest that the *nowl/LZTR1* gene encodes a conserved regulator of sleep that interacts with Nf1 and that may contribute to the overall pathophysiology of the 22q11.2 DS. Further studies of Nowl may help understand the molecular mechanisms underlying both mental disorders and Schwann-cell tumors.

## Materials and methods

### *Drosophila* lines and maintenance

*Drosophila* larvae and adults of mixed sexes were raised on standard cornmeal medium (Nutri-Fly “Bloomington” formulation) at 25°C under a 12:12-hour light/dark cycle with 60% humidity. The following fly lines were obtained from Bloomington *Drosophila* Stock Center (BDSC; Bloomington, IL): *elav-GAL4; UAS-Dicer-2* (#25750), *elav-GS-Gal4* (#43642), *nSyb-GAL4* (#51635), *Gad1-GAL4/CyO* (#51630), *Rdl-GAL4/CyO* (#66509), *Pdf-GAL4* (#6899), *dimm-GAL4* (#25373), *UAS-Dicer-2* (#24651), *Mi{ET1}CG3711*^*MB12128*^ (#29940), *Rdl*^*CB2*^*/TM6B*, *Tb*^*1*^ (#35493), *UAS-mCD8*::*GFP* (#4776), and *ppk-Gal4* (#32078). *UAS-RNAi* lines against orthologs of human 22q11.2 CNV-linked genes were ordered from Vienna *Drosophila* Resource Center (VDRC; Vienna, Austria) and are listed in S1 Table. *UAS-Cul3-RNAi* (#109415), *UAS-Nf1-RNAi* (#109637), and the *w*^*1118*^ (#60000) genetic background line were also obtained from VDRC.

The *GABA-B-R2*-*GAL4*::*p65* line carries a bacterial artificial chromosome (BAC) containing ~80 kb of genomic sequence surrounding the *GABA-B-R2* gene, in which the first coding exon of this gene was replaced with *GAL4*::*p65* and associated terminator sequences; the remaining exons and introns are still present, with the assumption that they contain regulatory sequences, but they are no longer transcribed. This construct was built using recombineering techniques [71] in P[acman] BAC clone CH321-95N09 (Children’s Hospital Oakland Research Institute, Oakland, CA). Selectable markers (conferring kanamycin resistance and streptomycin sensitivity) were cloned from *pSK+KanaRpsL* [72] (AddGene plasmid #20871) and flanked with upstream *GAL4* and downstream *HSP70-UTR* arms cloned from *pBPGUw* [73], a gift of G. Rubin. *GABA-B-R2*-specific homology arms were added by PCRing this landing-site cassette with the following primers (genomic sequence is in lower case): GABA-B-R2-GAL4-F: cgatatgcgc tattcacatt tagaatcgtt ttacagccca cgcggtcaac ATGAAGCTAC TGTCTTCTAT CGAACAAGC; GABA-B-R2-UTR-R: catcatcaga gattcactta atgaaatctt caagctaaac cctaactcac GATCTAAACG AGTTTTTAAG CAAACTCACT CCC. The homology-flanked landing-site cassette was recombined into the BAC (with kanamycin selection), and then full-length *GAL4*::*p65-HSP70* amplified from *pBPGAL4*.*2*::*p65Uw* [74] (a gift of G. Rubin) was recombined into this site in a second recombination (with streptomycin selection). The recombined regions of the BAC were sequenced, and the sequence-verified BAC was integrated into the *Drosophila* genome at *attP40* [75] by Genetic Services, Inc. (Cambridge, MA).

### Generation of CRISPR mutant

The CRISPR/Cas9 method was used to disrupt the *nowl* locus. Two guide RNAs (gRNAs) were designed using the Shigen Cas9 Target Finder (https://shigen.nig.ac.jp/fly/nigfly/cas9) and checked for potential off-target sites using FlyCRISPR Optimal Target Finder tool (http://targetfinder.flycrispr.neuro.brown.edu/). One gRNA was designed to target exon 1 and the second one to target exon 6, thus removing almost the entire *nowl* open reading frame. Oligonucleotides containing these gRNA sequences (gRNA1: CTT CGT GCT GGG CGT CTA AGC AGC; gRNA2: CTT CGA CCT AGA GTA GGA TTC ATC), designed with complementary *Bbs1* restriction-site overhangs, were cloned into the *pBFvU6*.*2* vector. Four μl of the forward and reverse oligos (100 μM) were annealed in 2 μl of 5x Phusion PH Buffer by heating the reaction at 95°C for 5 min and then leaving it to cool down to room temperature. One microgram of the *pBFv-U6*.*2* vector was cut using *BbsI* enzyme at 37°C for 1 h, and the enzyme was inactivated by raising the temperature to 65°C for 20 min. The cut vector was purified through gel electrophoresis, and the QIAquick gel-extraction kit (QIAGEN) was used to extract the DNA. The annealed oligonucleotides were then ligated into cut *pBFv-U6*.*2*. One μl of the digested vector, 1 μl of annealed oligonucleotides, 0.5 μl of T4 ligase, and 2 μl of 10X T4 ligase buffer (Thermo Scientific) was mixed to a final volume of 10 μl. For ligation, the reactions were incubated for 1 h at 37°C and transformed into 50 μl of Stellar competent cells (Clonetech) that were grown overnight at 37°C on LB plates containing ampicillin (100 μg/ml). Several colonies were selected and grown into liquid culture overnight. Colony PCR was conducted to identify the colonies containing properly configured plasmid using one gRNA oligo in combination with the M13 reverse primer. Plasmid DNA was extracted and sequenced to confirm the correct insertion of gRNA sequence. Plasmids were then injected into *y*^*1*^*w*^*1118*^; *attP2(nos-Cas9)/TM6*, *Sb*, *Tb* embryos by BestGene Inc (Chino Hills, CA).

### Identification of *Drosophila* orthologs

*Drosophila* orthologs of human 22q11.2 CNV-linked genes were identified using the “*Drosophila* RNAi Screening Center Integrative Ortholog Prediction Tool” (DIOPT), available at the website of the *Drosophila* RNAi Screening Center (DRSC), Harvard Medical School [22].

### Sleep assays and analysis

The *Drosophila* Activity Monitor (DAM) system (TriKinetics, Inc., Waltham, MA) was used to measure locomotion. Adult flies were sorted by sex and collected in groups of 30 soon after eclosion. Collected flies were housed under standard conditions until the start of the experiment. Three-to-seven-day-old flies (either male only or virgin female only, as indicated) were used for experiments, during which they were housed in 65-mm-long glass tubes containing a plug of food medium (5% sucrose and 2% agar in water) at one end and a cotton stopper at the other. For experiments using the GeneSwitch system, newly eclosed adult flies were maintained on normal food containing 1 mM RU486 (Sigma #M8086) dissolved in ethanol, or normal food containing a similar amount of ethanol, for 5–7 days. Flies were then loaded into tubes containing 5% sucrose and 2% agar in water with or without 1 mM RU486 in ethanol. Control flies were fed equal amounts of ethanol in their food. Experiments were run in a behavioral incubator under a 12-hour light/12-hour dark cycle, and flies were allowed 12–24 hours to acclimatize prior to experimental start. Activity, determined as the number of beam crosses per fly per minute, was measured in one-minute periods, and episodes of sleep were defined as at periods of least 5 minutes of uninterrupted quiescence. Flies exhibiting less than 10 minutes of activity during either the entire light or dark phase were flagged as dead. The pySolo software package [76] and a custom MATLAB script (MATLAB R2016b, The MathWorks Inc, Natick, Massachusetts) were used to analyze sleep dynamics. Sleep latency was calculated as the period from lights-off to the first sleep episode. Locomotor activity of male flies kept on a normal LD cycle for several days was monitored in constant darkness (DD) for 8 days to determine the length of the free-running circadian period. Data were aggregated into 30-minute bins and analyzed using the FaasX software package [77], which uses Chi-Square tests to calculate the period length. For mechanical sleep deprivation, flies in DAM monitors were stimulated using a vortexer plate (TriKinetics) for 2 seconds every minute during a 6-hour period before lights-on. Recovery sleep was determined during the first three hours following sleep deprivation.

### Western blotting

For Western-blot analysis, ten male flies per genotype were frozen at -80°C, vortexed, and sieved to obtain samples of heads. Heads were homogenized in 50 μl Laemmli Sample Buffer (Bio-Rad) containing 2-mercaptoethanol. Samples were denatured for 5 minutes at 95°C and then centrifuged at maximum speed (17,000 g) for 5 minutes. Samples (20 μl) were loaded on a 4–20% gradient polyacrylamide gel (Bio-Rad), and proteins were separated at 150 V for 30 minutes. Proteins were transferred onto PVDF membrane (Millipore), and the membrane was blocked for one hour in Odyssey Blocking Buffer (LI-COR). Primary antibodies (rabbit anti-phospho-ERK, Cell Signaling Technology #9101, 1:1000; mouse anti-α-tubulin, Sigma-Aldrich #T9026, 1:5000) were diluted in Odyssey Blocking Buffer containing 0.2% Tween 20, and the membrane was incubated overnight in this solution. Membranes were then rinsed and incubated for 40 minutes with secondary antibodies conjugated to infrared fluorophores (anti-mouse IRDye 680RD and anti-rabbit 800CW, LI-COR, 1:10,000), and staining was imaged using an Odyssey Fc scanner (LI-COR).

### Anti-Nowl antiserum development, immunostaining and class IV neuron visualization

To generate a Nowl antibody, a peptide corresponding to residues 123–141 of the protein (LRSSFKSSKRNKARKSAST) was used in a custom immunization protocol carried out by Genosphere Biotechnologies (Paris, France). Epitope specificity of the antiserum was confirmed by comparing wild-type (*Rdl>GFP*) and *elav>nowl-RNAi* animals by immunostaining (S9C Fig) as well as by co-application of the pre-immune serum. For immunocytochemistry, primary antibodies used were polyclonal rabbit α-Nowl (1:200), α-phospho-Histone H3 (αPH3; 1:500; EMD Millipore #06–570), and FITC-conjugated polyclonal goat anti-GFP (1:200; Abcam, #ab6662). Biotinylated anti-rabbit was applied (1:200; Vector Laboratories, #BA-1100), followed by Cy3-streptavidin (1:200; Jackson ImmunoResearch Laboratories, #016-160-084). Tissues were mounted on poly-L-lysine-coated 35-mm glass-bottomed dishes (MatTek Corporation, MA, USA) in Vectashield (Vector Laboratories Inc., CA, USA), and image acquisition was performed on an inverted Zeiss LSM800 confocal microscope with AiryScan (Zeiss, Oberkochen, Germany). Images were processed in CorelDraw X8 (Corel, Ottawa, Canada). For visualization of class-IV da neurons, wandering third-instar larvae expressing GFP driven by the class-IV-specific *ppk>* driver were anesthetized by exposure to ether for five minutes in a sealed container. For visualization of dendritic arbors, live larvae were mounted in 90% glycerol and imaged for GFP. Imaging of class-IV axonal terminals was performed on dissected brains with ventral nerve cords, mounted in Vectashield.

### Transcript analysis by quantitative PCR

The efficiency of *nowl* and *Nf1* RNAi-mediated gene knockdown was analyzed using quantitative RT-PCR (qPCR). Heads from adult male flies expressing neuronal RNAi against *nowl* or *Nf1*, along with driver- and RNAi-alone controls, were collected, and total RNA was extracted from 10 heads per sample using the RNeasy Mini Kit (Qiagen) according to the manufacturer’s instructions. cDNA was reverse-transcribed from total RNA using the High-Capacity cDNA Reverse Transcription Kit (ThermoFisher #4368814) and used as a template for qPCR with the QuantiTect SYBR Green PCR Kit (Fisher Scientific #204145) and an Mx3005P qPCR System (Agilent Technologies). Levels of target-gene expression were normalized against *RpL32*. Precomputed oligo pairs from the FlyPrimerBank were used: GGAGGATCGGGATGTAGTATGG and CGTGTAGGAGGTGAAGTCCAC for *nowl*; AGTATCTGATGCCCAACATCG and CAATCTCCTTGCGCTTCTTG for *Nf1*; and CTTTTGGCACGTTTCGAGGAT and GGTAGCGCGATATGTGGATCAG for *RpL32*.

### Measurements of glycogen and triglycerides

For triglyceride and glycogen assays, one-week-old adult males were collected and frozen at -80 ^o^C in groups of three animals. Animals were homogenized in 80 μl PBS + 0.5% Tween using a Tissue Lyser LT (Qiagen) with small steel beads. For triglyceride measurements, 8 μl of the homogenate was added to 80 μl Free Glycerol Reagent (Sigma, F6428) and 20 μl Triglyceride Reagent (Sigma, T2449), and reactions were incubated at 37 ^o^C for 10 minutes with agitation, before being read at 540-nm absorbance. For glycogen measurements, 6 μl homogenate was mixed with 94 μl Glucose Oxidase (GO) reagent from the Glucose (GO) Assay Kit (Sigma, GAGO20) containing 1 μl (0.3 U) of low-glucose Amyloglucosidase (Sigma, A7420-5MG) to determine total glucose + glycogen. Another 6 μl homogenate was mixed with 94 μl GO reagent alone (without the Amyloglucosidase) to determine free glucose. For analysis of glycogen content of heads, each sample was based on 5 adult heads homogenized in 40 μl PBS + 0.5% Tween, of which 4 μl homogenate was used with 25 ul GO reagent with and without the Amyloglucosidase. Reactions were incubated at 37 ^o^C for 20 minutes, after which 12-N sulfuric acid was added to stop and complete the reaction (66 μl for whole-body and 12 μl for head samples) before absorbance was measured at 540 nm. Absorbance was measured using an EnSight multimode plate reader (PerkinElmer), and values were standardized against readings for a range of known concentrations.

### Statistics

GraphPad Prism software was used for statistical analysis. For single comparisons, significance was analyzed by Mann-Whitney U tests. When comparing multiple samples, Kruskal-Wallis tests with Dunn's post-hoc testing were used. Statistical significance was set at p≤0.05.

## Supporting information

S1 Fig Screening individual *Drosophila* orthologs of genes spanned by the human 22q11.2 deletion for effects on sleep and activity in adult females. (A, B) Quantification of total sleep (A) and average activity measured as the number of beam crosses per minute (B) in 3-to-7-day-old females, measured over a 24-hour period. *UAS-RNAi* constructs were expressed under the control of the pan-neuronal *elav-GAL4* driver. n = 140 flies for controls (*elav>* crossed to *w*^*1118*^, the genetic background for the RNAi lines); n = 16 flies for each RNAi genotype. When possible, two distinct *UAS-RNAi* constructs were used against each gene. RNAi efficiency was enhanced by co-expressing the processing enzyme Dicer-2 (*UAS-Dcr-2*). Graphs represent means with SEM of data pooled from one to five independent experiments. Statistical significance was determined using Kruskal-Wallis test with Dunn's post-hoc testing (* p<0.05).
(PDF)

S2 Fig The CRISPR-induced *nowl*^*KO*^ mutation causes a sleep phenotype similar to that seen with the *nowl*^*1*^ insertion allele. (A) Daily sleep profiles across a 12-hour light, 12-hour dark (white and black bars) cycle for 3-to-7-day-old male controls (*w*^*1118*^/Y) compared to *nowl*^*KO*^ mutants (*nowl*^*KO*^/Y). Sleep data are binned into 30-minute intervals. (B, C) Total day- and night-time sleep (minutes) in males. (C) Total night-time sleep is decreased in *nowl*^*KO*^ mutants. (D) Sleep latency in males in *nowl*^*KO*^ mutant males is increased compared to the control. (E-H) Quantification of average sleep-bout duration and number during day and night for male *nowl*^*KO*^ mutants compared to controls. (G, H) Night-time sleep is fragmented in *nowl*^*KO*^ mutant males compared to the control, as indicated by increased sleep-bout number (G) and reduced sleep-bout duration (H). Graphs represent mean and SEM (n = 32–75) of data pooled from one to three experiments. Significance was tested by Mann-Whitney U tests (*** p < 0.001).
(PDF)

S3 Fig *nowl* mutants exhibit normal circadian period with a weaker circadian clock. (A, B) Circadian rhythmicity of 3-to-8-day-old male flies kept in the dark for 6 days. During entrainment, lights-on occurred at Zeitgeber time (ZT) 0 and lights-off occurred at ZT12. (A) Actograms showing average activity during the light/dark entrainment period and during constant-darkness free-running cycles (ZT in hours). (B) Representative periodograms of *nowl*^*1*^ mutants indicate that it cycles similarly to controls (*w*^*1118*^/Y and +/Y) with a nearly 24-hour period, indicating that *nowl* is not necessary for entrainment or free running of the circadian clock. The data in the periodograms begins 24 hours after lights-OFF transition. (C and D) Strength of the clock and rhythmicity of male flies. (C) Representative graphs of individual rhythmic flies showing the strength of circadian clock which is defined by the main peak (the power is the height of the peak above the 5% significance level. (D) Quantification of the percentage of rhythmic flies, the average period, and the strength of rhythmicity (the power and the width of the peak, an additional measure of the strength of the circadian clock). The chi-squared analysis shows that the main peak is lower for *nowl*^*1*^ mutants compared to one of the controls. Similarly, the width of the peak is lower for *nowl*^*1*^ mutants than controls. Only the data of rhythmic flies were taken into account for data in (B-D). Data represents means (n = 17–28) of data from one experiment and was analysed by chi-square test in the FaasX software package [77].
(PDF)

S4 Fig Screening individual *Drosophila* orthologs of human 22q11.2 deletion genes for effects on sleep maintenance. Neuronal gene knockdown was induced by crossing *elav>* to each gene-specific *UAS-RNAi* line. As a control, the *elav>* line was crossed to *w*^*1118*^. *(*A-D) Sleep-episode duration and number during the day (A, B) and night (C, D) of 3-to-8-day-old male flies (n = 140 flies for controls; n = 16 flies for each RNAi genotype). Graphs represent mean and SEM of data pooled from one to five independent experiments. Significance was determined using Kruskal-Wallis test with Dunn's post-hoc testing (*p < 0.05).
(PDF)

S5 Fig Temporal sleep distribution for males with neuronal knockdown of *nowl* using *nSyb-GAL4*. (A) Daily sleep profiles across a 12-hour light, 12-hour dark (white and black bars) cycle for 3-to-7-day-old male controls (*nSyb>+* and *nowl-RNAi/+*) compared to *nSyb>nowl-RNAi* animals with knockdown of *nowl* in the nervous system. Sleep data are binned into 30-minute intervals. (B, C) Quantification of total day- and night-time sleep (minutes) in males. Graphs represent mean and SEM (n = 32) of data from one experiment.
(PDF)

S6 Fig Pan-neuronal knockdown of *Cul3* causes sleep fragmentation. (A) Daily sleep profiles across a 12-hour light, 12-hour dark (white and black bars) cycle for 3-to-7-day-old male controls (*elav>+* and *Cul3-RNAi/+*) compared to *elav>Cul3-RNAi* animals with knockdown of *Cul3* in the nervous system. (B, C) Quantification of average male daytime sleep-bout duration (B) and number (C) for *elav>Cul3-RNAi* flies compared to controls (*elav>+* and *UAS-Cul3-RNAi/+*). Sleep-bout length was significantly decreased, and sleep-bout number was significantly increased during the day when *Cul3* was knocked down in the nervous system. (D, E) Quantification of average night-time sleep-bout duration (D) and number (E) in males. Bout length was significantly decreased, and average bout numbers were significantly increased during the night upon pan-neuronal knockdown of *Cul3*. Graphs represent mean and SEM (n = 32) of data from one experiment, which is representative of two independent experiments. Statistical significance was determined using Kruskal-Wallis test with Dunn's post-hoc testing (*** p<0.001).
(PDF)

S7 Fig Knockdown of *nowl* and *Nf1* is not associated with proliferation or tumors in the central and peripheral nervous system. (A) Representative images of anti-phospho-Histone H3 (α-PH3, red) and DAPI (blue) staining of adult brains of 5-7-day-old males show absence of cell proliferation in animals with knockdown of *nowl* or *Nf1*. Cell proliferation, as indicated α-PH3, was not observed in analysis of five independent brains of each genotype. (B) Representative images of five independent class-IV dendritic arborization (da) neurons from abdominal segment A3 in the peripheral nervous system and their axonal terminals in the ventral nerve cord. Knockdown of *nowl* or *Nf1* does not alter the gross morphology of class-IV da neuronal structures.
(PDF)

S8 Fig Effector line controls related to main Fig 5. Quantification of daytime (A) and nighttime (B) total sleep, sleep-bout duration, and sleep-bout numbers for *nowl-RNAi/+*, *Nf1-RNAi/+* and *UAS-Nf1/+* (*Nf1/+*) control males for main Fig 5. Graphs represent means with SEM (n = 32–81) of data pooled from one to three independent experiments.
(PDF)

S9 Fig RNAi against *nowl* and *Nf1* is effective, and Nowl antibody staining is specific. (A, B) Efficiency of *nowl* (A) and *Nf1* (B) gene knockdown. Gene expression was determined in adult male heads using qPCR. (C) Anti-Nowl staining features observed in male *Rdl>GFP* controls are not present in *elav>nowl-RNAi* brains with reduced expression of *nowl* in the nervous system, indicating that the anti-Nowl antibody specifically recognizes the Nowl protein. Graphs represent mean and SEM (n = 6) of data from one experiment. Statistical significance was determined using Kruskal-Wallis test with Dunn's post-hoc testing (*p < 0.05, *** p < 0.001).
(PDF)

S10 Fig Adult-specific knockdown of *nowl* and *Nf1* leads to reduced and fragmented night-time sleep phenotypes. RU486 was used to induce RNAi restricted to the adult stage using the GeneSwitch (GS) system in flies carrying *elav-GS-GAL4* (*elavGS*>). (A, B) Total sleep per night is decreased in male flies with adult-specific neuronal knockdown of *nowl* (*elavGS>nowl-RNAi*) or *Nf1* (*elavGS>Nf1-RNAi*) induced by RU486 compared to vehicle-treated controls and *elavGS>+* controls. (C-F) Night-time sleep is fragmented in adult males when knockdown of *nowl* or *Nf1* is induced, as indicated by reduced sleep-bout duration (C, D) and increased sleep-bout number (E, F) primarily during the night. Graphs represent mean and SEM (n = 32) of data from one experiment. Significance was tested by Mann-Whitney U tests (*p < 0.05, ** p < 0.01, *** p < 0.001).
(PDF)

S11 Fig Increased GABA signaling rescues the sleep phenotype of *nowl* mutants. Daily sleep profiles across a 12-hour light, 12-hour dark (white and black bars) cycle for 3-to-7-day-old male flies of the genotypes *w*^*1118*^*/Y*, *nowl*^*1*^*/Y*, *Rdl*^*CB2*^*/+*, and *nowl*^*1*^*/Y; Rdl*^*CB2*^*/+*. The reduced night-time sleep produced by loss of *nowl* (*nowl*^*1*^ mutant) is rescued by introducing one copy of the *Rdl*^*CB2*^ allele.
(PDF)

S1 Table Transgenic UAS-RNAi fly lines targeting orthologs of 22q11.2-linked genes. (DOCX)
