# Supplementary figures and images for: Analysis of genes within the schizophrenia-linked 22q11.2 deletion identifies interaction of night owl/LZTR1 and NF1 in GABAergic sleep control
Source: PLoS Genet. 2020 Apr 27;16(4):e1008727. doi: 10.1371/journal.pgen.1008727 (PMC7205319; doi:10.1371/journal.pgen.1008727)

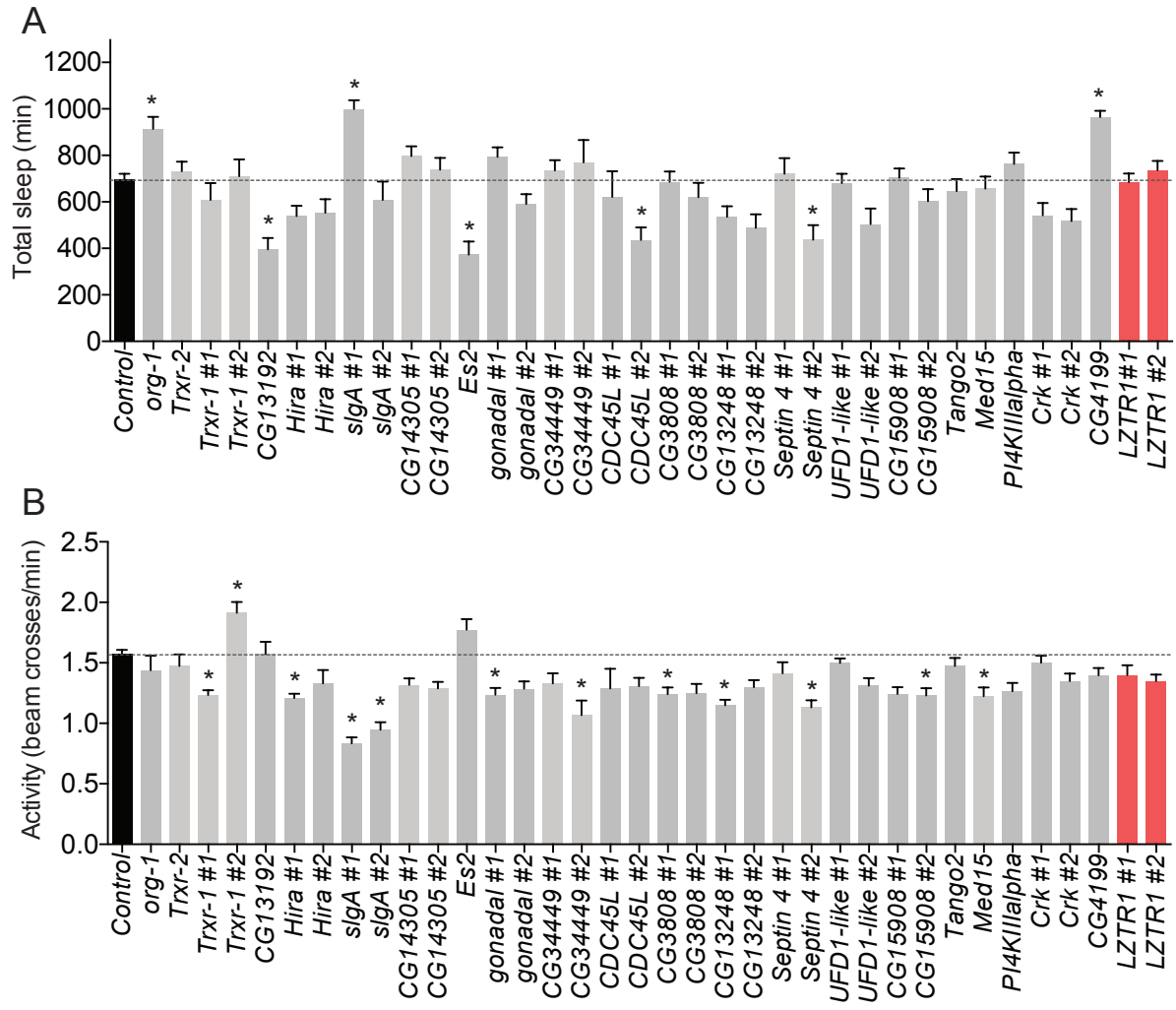

Supplement: S1 Fig — (A, B) Quantification of total sleep (A) and average activity measured as the number of beam crosses per minute (B) in 3-to-7-day-old females, measured over a 24-hour period. UAS-RNAi constructs were expressed under the control of the pan-neuronal elav-GAL4 driver. n = 140 flies for controls (elav> crossed to w1118, the genetic background for the RNAi lines); n = 16 flies for each RNAi genotype. When possible, two distinct UAS-RNAi constructs were used against each gene. RNAi efficiency was enhanced by co-expressing the processing enzyme Dicer-2 (UAS-Dcr-2). Graphs represent means with SEM of data pooled from one to five independent experiments. Statistical significance was determined using Kruskal-Wallis test with Dunn's post-hoc testing (* p<0.05). (PDF) [file pgen.1008727.s001.pdf]

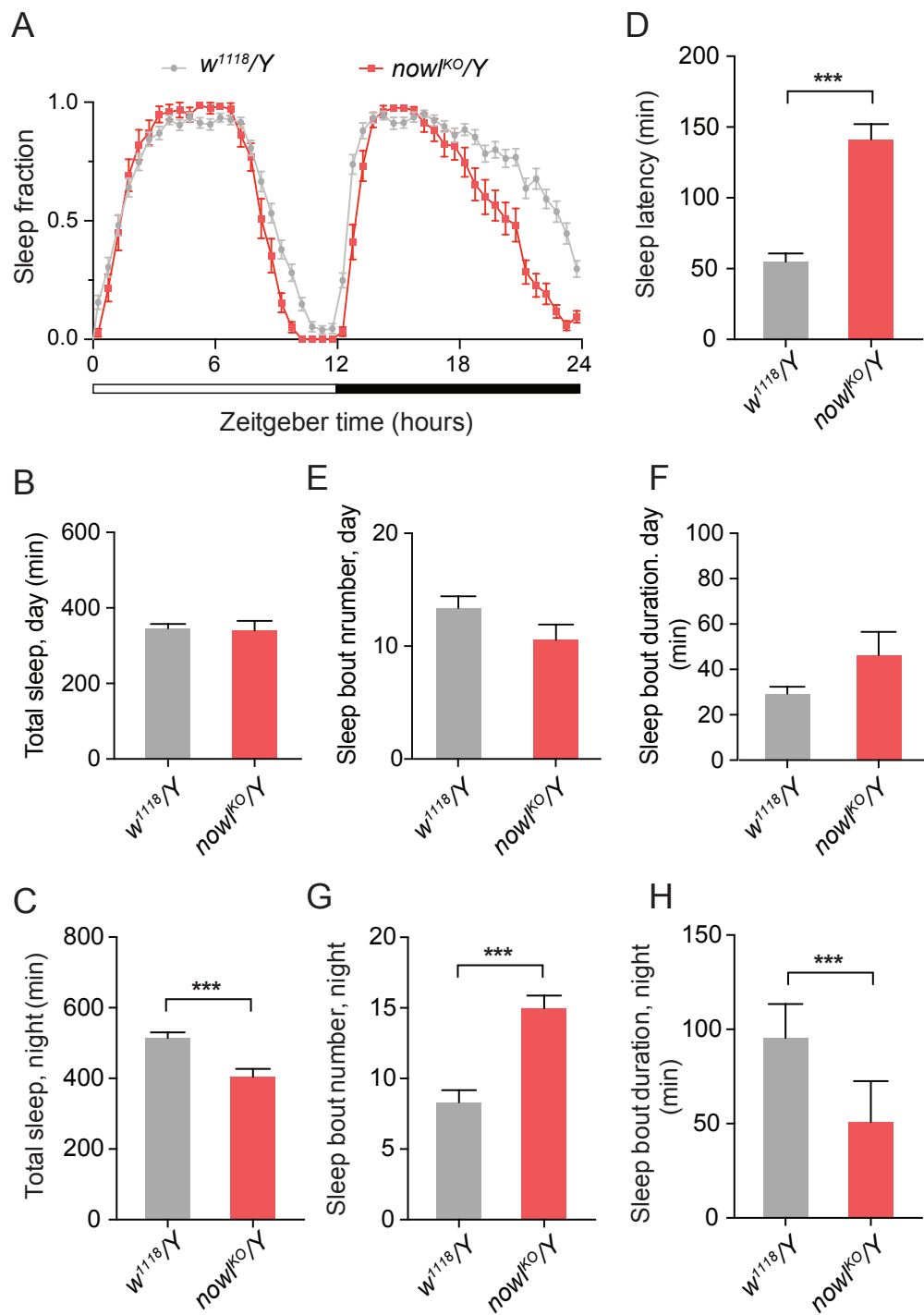

Supplement: S2 Fig — (A) Daily sleep profiles across a 12-hour light, 12-hour dark (white and black bars) cycle for 3-to-7-day-old male controls (w1118/Y) compared to nowlKO mutants (nowlKO/Y). Sleep data are binned into 30-minute intervals. (B, C) Total day- and night-time sleep (minutes) in males. (C) Total night-time sleep is decreased in nowlKO mutants. (D) Sleep latency in males in nowlKO mutant males is increased compared to the control. (E-H) Quantification of average sleep-bout duration and number during day and night for male nowlKO mutants compared to controls. (G, H) Night-time sleep is fragmented in nowlKO mutant males compared to the control, as indicated by increased sleep-bout number (G) and reduced sleep-bout duration (H). Graphs represent mean and SEM (n = 32–75) of data pooled from one to three experiments. Significance was tested by Mann-Whitney U tests (*** p < 0.001). (PDF) [file pgen.1008727.s002.pdf]

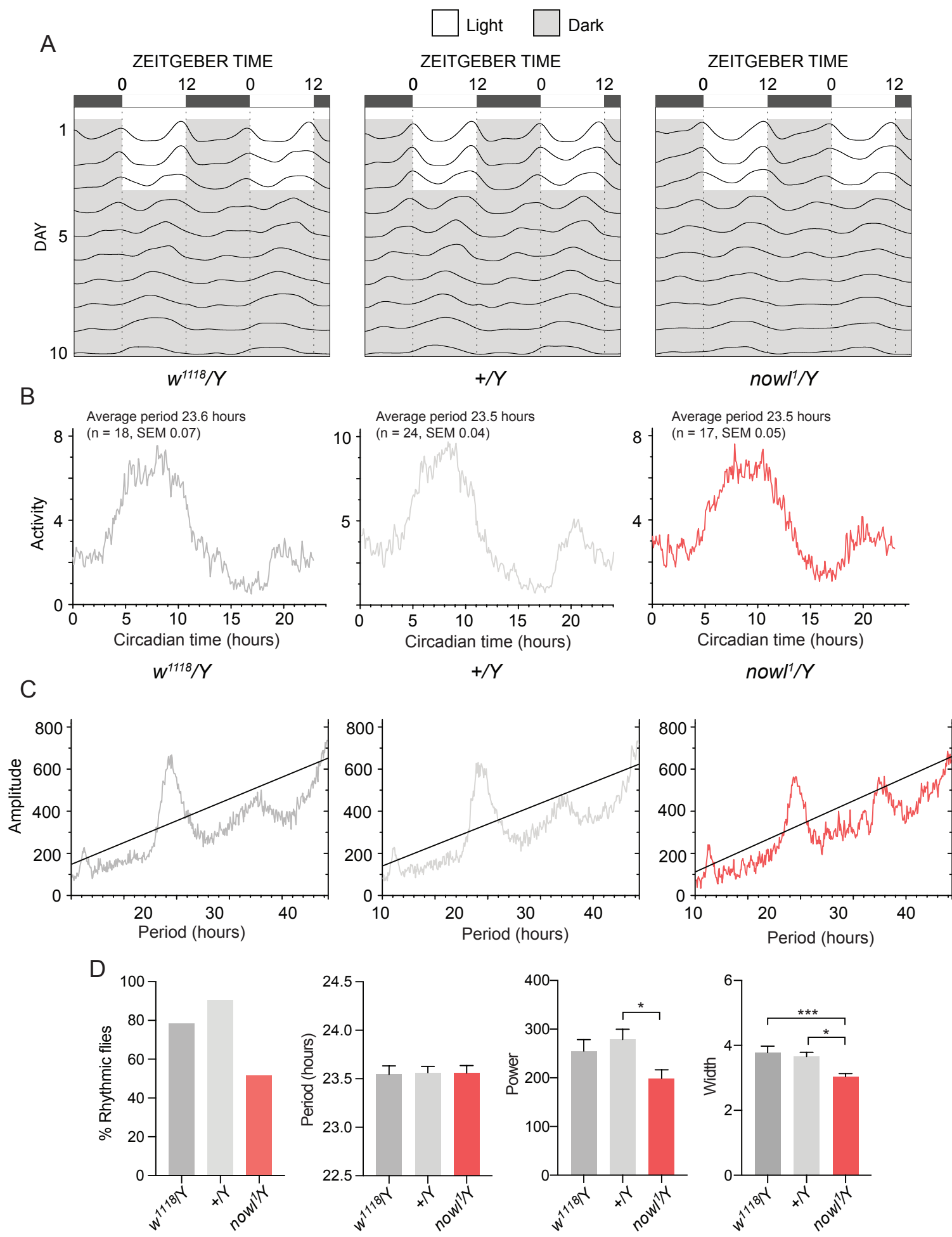

Supplement: S3 Fig — (A, B) Circadian rhythmicity of 3-to-8-day-old male flies kept in the dark for 6 days. During entrainment, lights-on occurred at Zeitgeber time (ZT) 0 and lights-off occurred at ZT12. (A) Actograms showing average activity during the light/dark entrainment period and during constant-darkness free-running cycles (ZT in hours). (B) Representative periodograms of nowl1 mutants indicate that it cycles similarly to controls (w1118/Y and +/Y) with a nearly 24-hour period, indicating that nowl is not necessary for entrainment or free running of the circadian clock. The data in the periodograms begins 24 hours after lights-OFF transition. (C and D) Strength of the clock and rhythmicity of male flies. (C) Representative graphs of individual rhythmic flies showing the strength of circadian clock which is defined by the main peak (the power is the height of the peak above the 5% significance level. (D) Quantification of the percentage of rhythmic flies, the average period, and the strength of rhythmicity (the power and the width of the peak, an additional measure of the strength of the circadian clock). The chi-squared analysis shows that the main peak is lower for nowl1 mutants compared to one of the controls. Similarly, the width of the peak is lower for nowl1 mutants than controls. Only the data of rhythmic flies were taken into account for data in (B-D). Data represents means (n = 17–28) of data from one experiment and was analysed by chi-square test in the FaasX software package [77]. (PDF) [file pgen.1008727.s003.pdf]

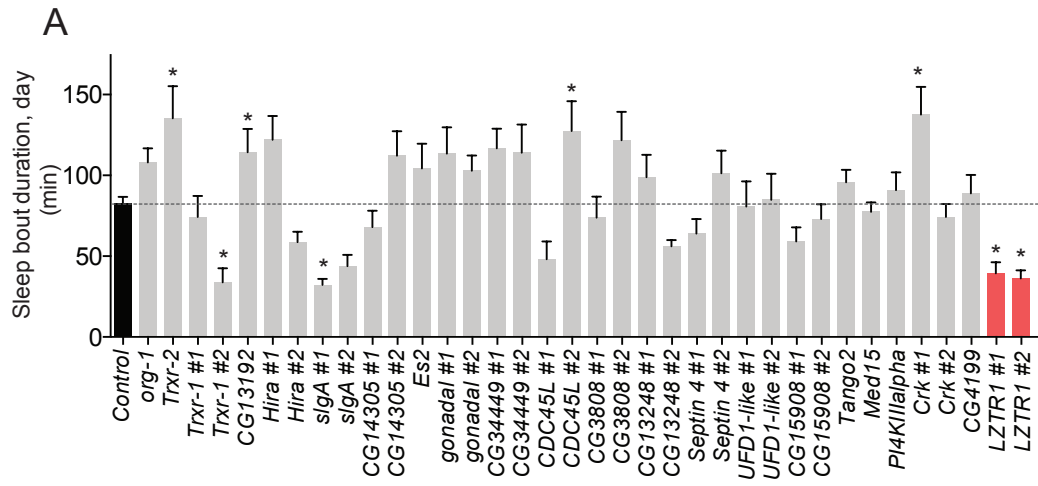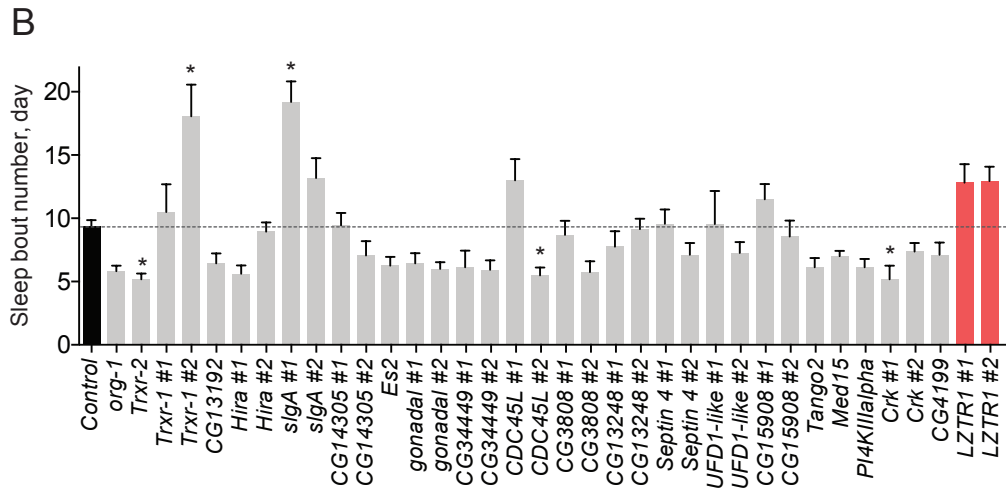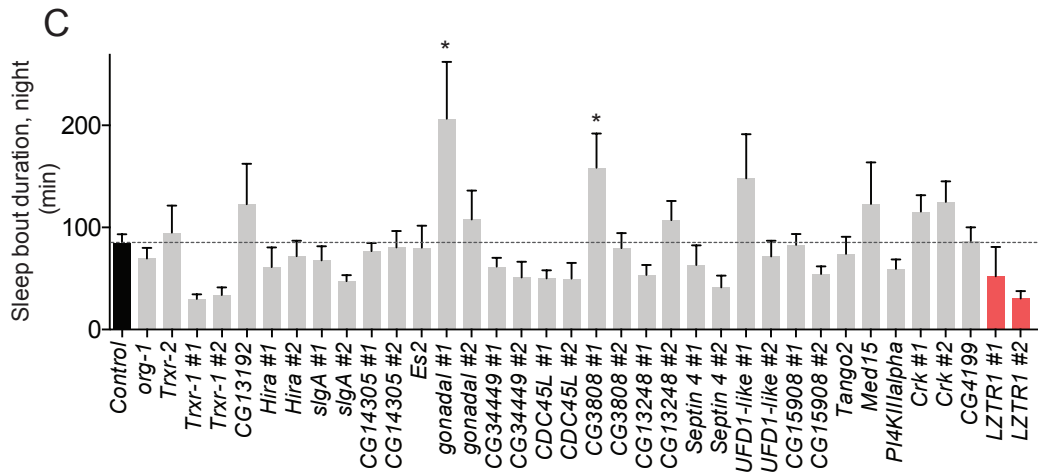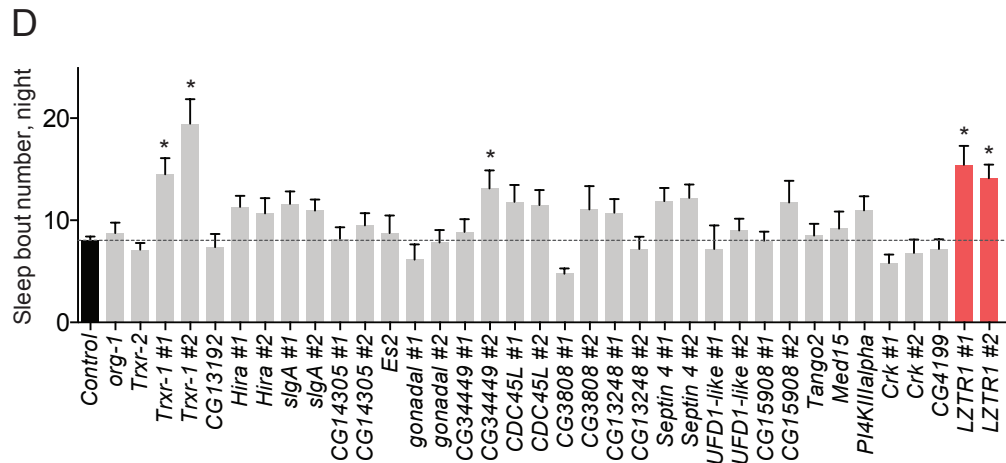

Supplement: S4 Fig — Neuronal gene knockdown was induced by crossing elav> to each gene-specific UAS-RNAi line. As a control, the elav> line was crossed to w1118. (A-D) Sleep-episode duration and number during the day (A, B) and night (C, D) of 3-to-8-day-old male flies (n = 140 flies for controls; n = 16 flies for each RNAi genotype). Graphs represent mean and SEM of data pooled from one to five independent experiments. Significance was determined using Kruskal-Wallis test with Dunn's post-hoc testing (*p < 0.05). (PDF) [file pgen.1008727.s004.pdf]

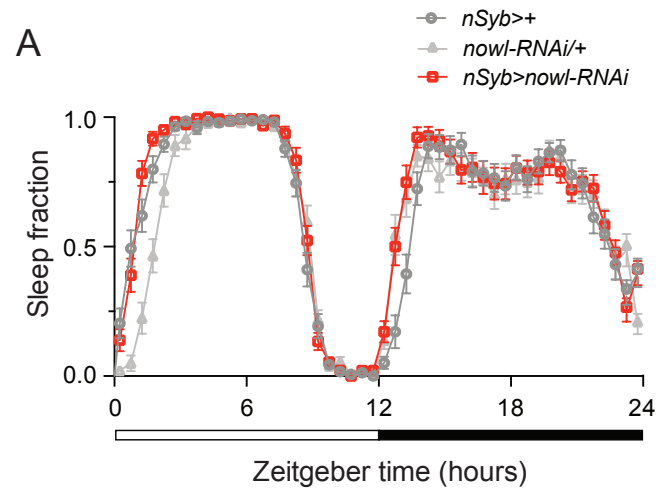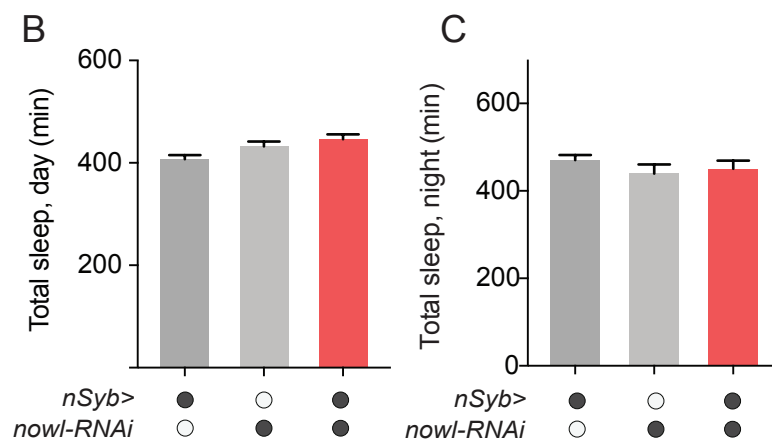

Supplement: S5 Fig — (A) Daily sleep profiles across a 12-hour light, 12-hour dark (white and black bars) cycle for 3-to-7-day-old male controls (nSyb>+ and nowl-RNAi/+) compared to nSyb>nowl-RNAi animals with knockdown of nowl in the nervous system. Sleep data are binned into 30-minute intervals. (B, C) Quantification of total day- and night-time sleep (minutes) in males. Graphs represent mean and SEM (n = 32) of data from one experiment. (PDF) [file pgen.1008727.s005.pdf]

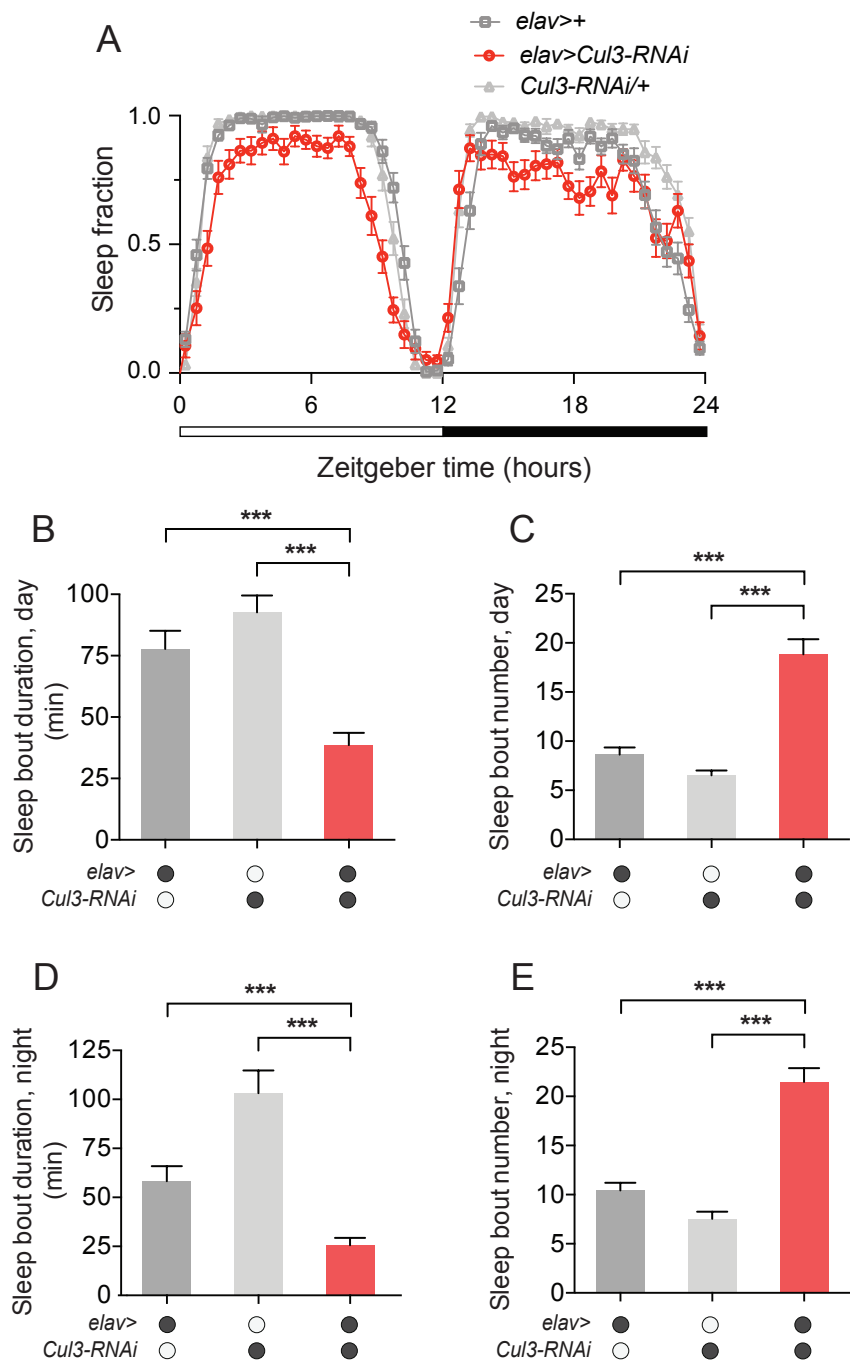

Supplement: S6 Fig — (A) Daily sleep profiles across a 12-hour light, 12-hour dark (white and black bars) cycle for 3-to-7-day-old male controls (elav>+ and Cul3-RNAi/+) compared to elav>Cul3-RNAi animals with knockdown of Cul3 in the nervous system. (B, C) Quantification of average male daytime sleep-bout duration (B) and number (C) for elav>Cul3-RNAi flies compared to controls (elav>+ and UAS-Cul3-RNAi/+). Sleep-bout length was significantly decreased, and sleep-bout number was significantly increased during the day when Cul3 was knocked down in the nervous system. (D, E) Quantification of average night-time sleep-bout duration (D) and number (E) in males. Bout length was significantly decreased, and average bout numbers were significantly increased during the night upon pan-neuronal knockdown of Cul3. Graphs represent mean and SEM (n = 32) of data from one experiment, which is representative of two independent experiments. Statistical significance was determined using Kruskal-Wallis test with Dunn's post-hoc testing (*** p<0.001). (PDF) [file pgen.1008727.s006.pdf]

A

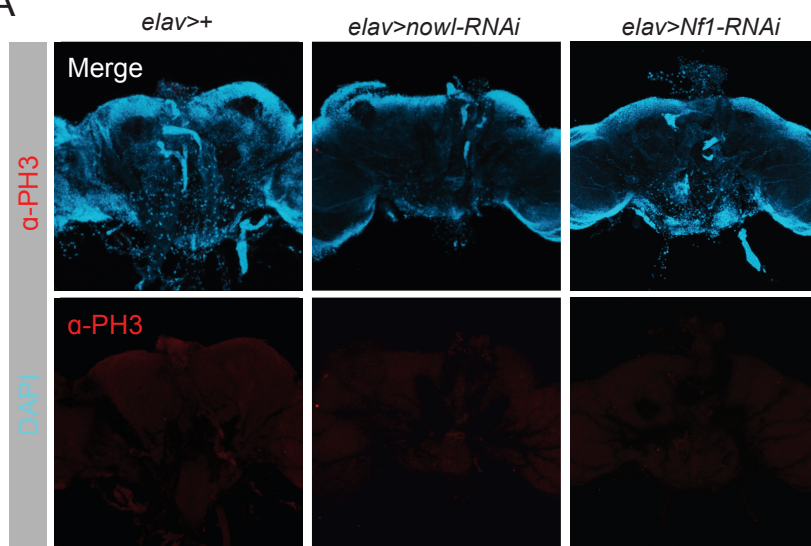

B

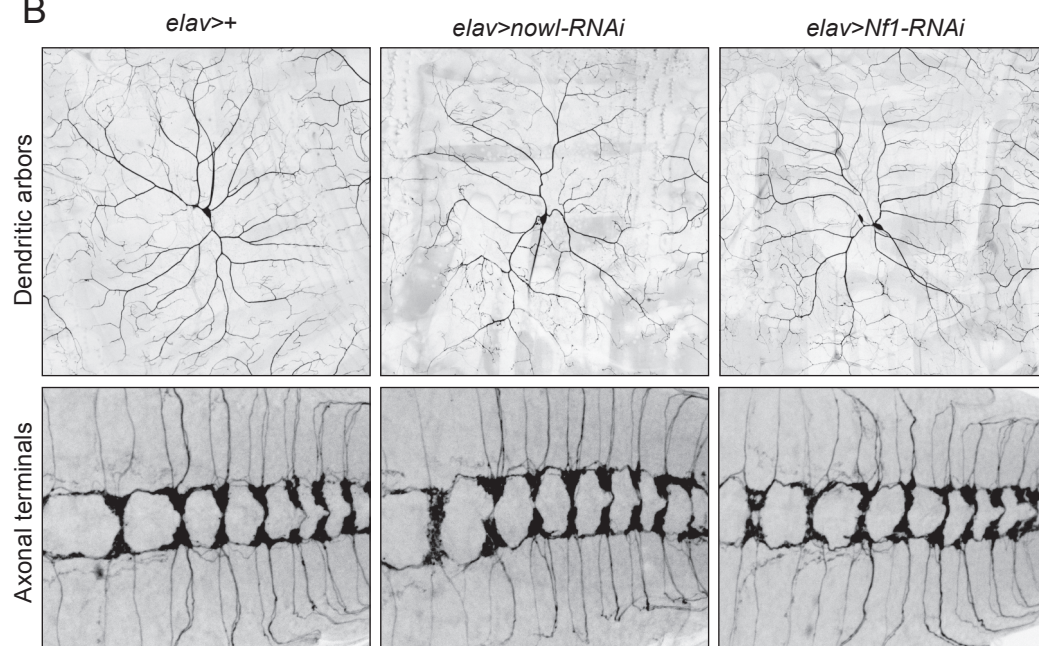

Supplement: S7 Fig — (A) Representative images of anti-phospho-Histone H3 (α-PH3, red) and DAPI (blue) staining of adult brains of 5-7-day-old males show absence of cell proliferation in animals with knockdown of nowl or Nf1. Cell proliferation, as indicated α-PH3, was not observed in analysis of five independent brains of each genotype. (B) Representative images of five independent class-IV dendritic arborization (da) neurons from abdominal segment A3 in the peripheral nervous system and their axonal terminals in the ventral nerve cord. Knockdown of nowl or Nf1 does not alter the gross morphology of class-IV da neuronal structures. (PDF) [file pgen.1008727.s007.pdf]

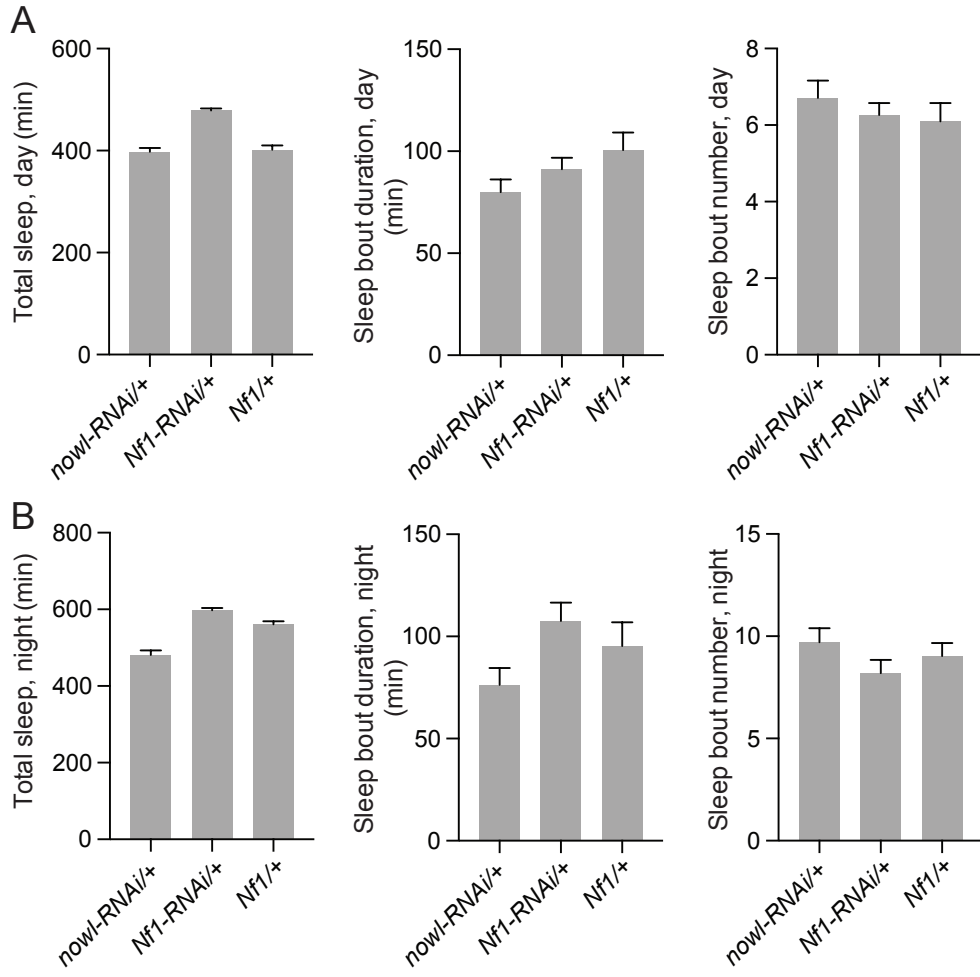

Supplement: S8 Fig — Quantification of daytime (A) and nighttime (B) total sleep, sleep-bout duration, and sleep-bout numbers for nowl-RNAi/+, Nf1-RNAi/+ and UAS-Nf1/+ (Nf1/+) control males for main Fig 5. Graphs represent means with SEM (n = 32–81) of data pooled from one to three independent experiments. (PDF) [file pgen.1008727.s008.pdf]

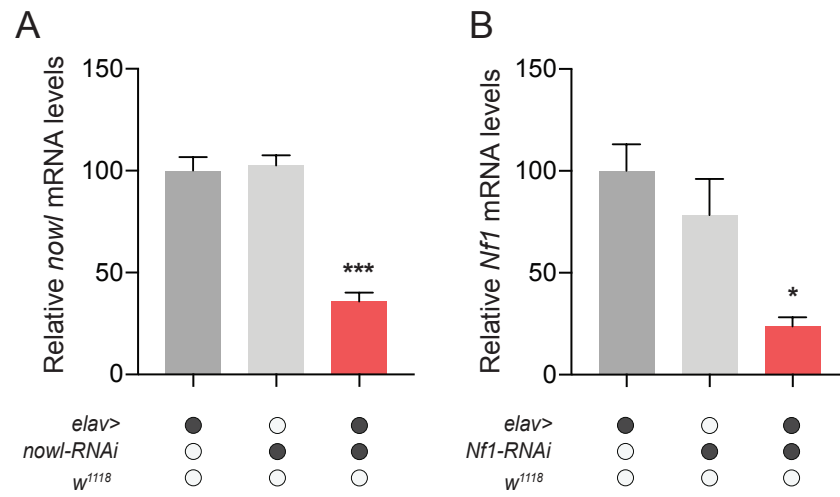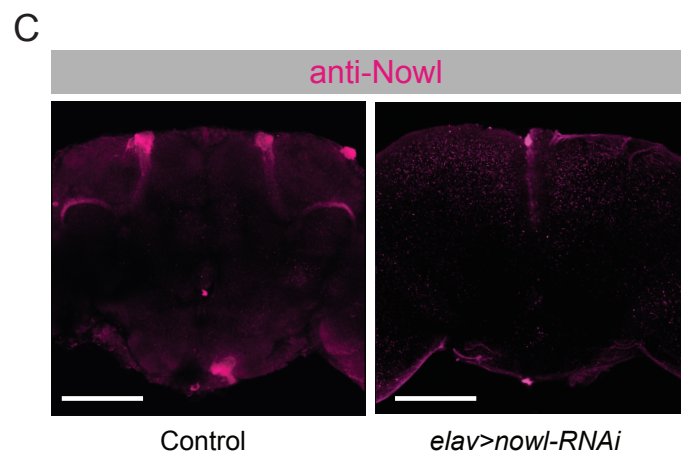

Supplement: S9 Fig — (A, B) Efficiency of nowl (A) and Nf1 (B) gene knockdown. Gene expression was determined in adult male heads using qPCR. (C) Anti-Nowl staining features observed in male Rdl>GFP controls are not present in elav>nowl-RNAi brains with reduced expression of nowl in the nervous system, indicating that the anti-Nowl antibody specifically recognizes the Nowl protein. Graphs represent mean and SEM (n = 6) of data from one experiment. Statistical significance was determined using Kruskal-Wallis test with Dunn's post-hoc testing (*p < 0.05, *** p < 0.001). (PDF) [file pgen.1008727.s009.pdf]

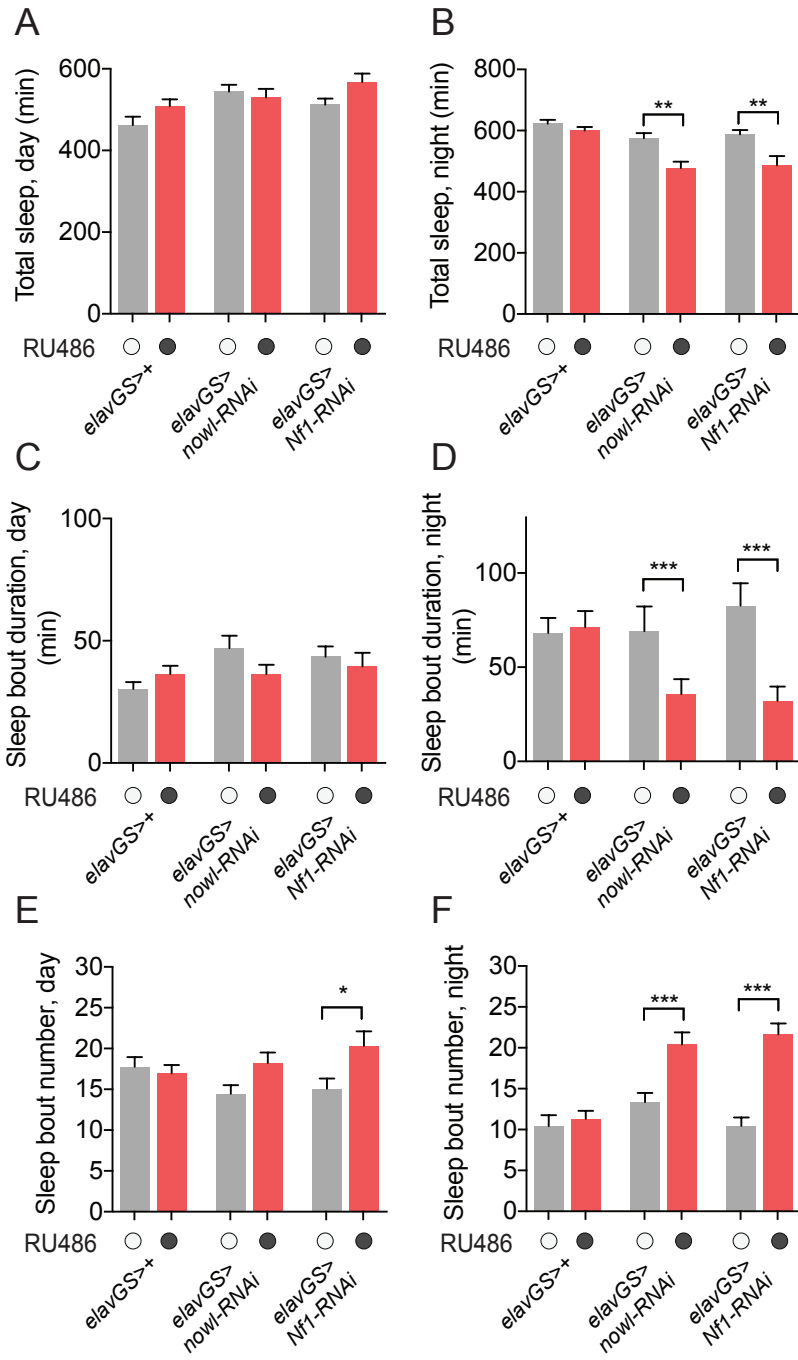

Supplement: S10 Fig — RU486 was used to induce RNAi restricted to the adult stage using the GeneSwitch (GS) system in flies carrying elav-GS-GAL4 (elavGS>). (A, B) Total sleep per night is decreased in male flies with adult-specific neuronal knockdown of nowl (elavGS>nowl-RNAi) or Nf1 (elavGS>Nf1-RNAi) induced by RU486 compared to vehicle-treated controls and elavGS>+ controls. (C-F) Night-time sleep is fragmented in adult males when knockdown of nowl or Nf1 is induced, as indicated by reduced sleep-bout duration (C, D) and increased sleep-bout number (E, F) primarily during the night. Graphs represent mean and SEM (n = 32) of data from one experiment. Significance was tested by Mann-Whitney U tests (*p < 0.05, ** p < 0.01, *** p < 0.001). (PDF) [file pgen.1008727.s010.pdf]

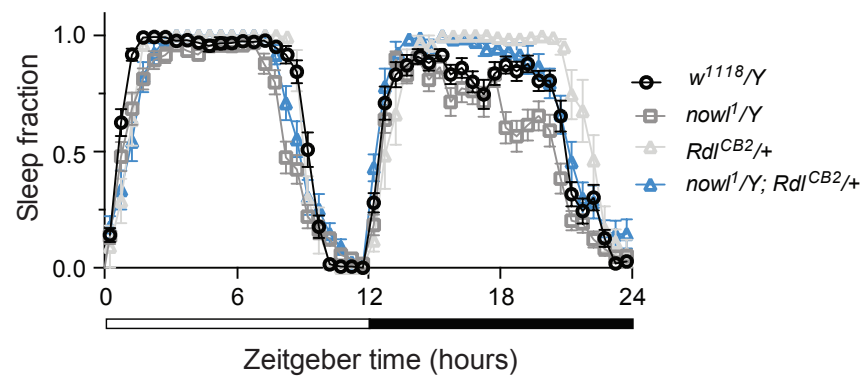

Supplement: S11 Fig — Daily sleep profiles across a 12-hour light, 12-hour dark (white and black bars) cycle for 3-to-7-day-old male flies of the genotypes w1118/Y, nowl1/Y, RdlCB2/+, and nowl1/Y; RdlCB2/+. The reduced night-time sleep produced by loss of nowl (nowl1 mutant) is rescued by introducing one copy of the RdlCB2 allele. (PDF) [file pgen.1008727.s011.pdf]
