# Supplementary material for: Analysis of genes within the schizophrenia-linked 22q11.2 deletion identifies interaction of night owl/LZTR1 and NF1 in GABAergic sleep control
Source: PLoS Genet. 2020 Apr 27;16(4):e1008727. doi: 10.1371/journal.pgen.1008727 (PMC7205319; doi:10.1371/journal.pgen.1008727)
Supplement: S1 Table — (DOCX) [file pgen.1008727.s012.docx]

| **Human gene** | ***Drosophila* ortholog** | **Fly line (*UAS-RNAi*) from VDRC** |
| --- | --- | --- |
| *PRODH* | *sluggish A* (*slgA*) | #18561 (#1)  #101449 (#2) |
| *TSSK2* | *CG14305* | #17477 (#1) #17478 (#2) |
| *DGCR14* | *Es2* | #42996 (#1)  #106243 (#2) |
| *SLC25A1* | *scheggia* (*sea*) | #50713 (#1)  #109169 (#2) |
| *CLTCL1* | *Clathrin heavy chain* (*Chc*) | #23666 (#1)  #103383 (#2) |
| *HIRA* | *Hira* | #13690 ( #1)  #106989 (#2) |
| *MRPL40* | *mRpL40* | #48166 (#1)  #101442 (#2) |
| *C22orf39* | *CG15908* | #19621 (#1)  #106340 (#2) |
| *UFD1L* | *Ubiquitin fusion-degradation 1-like* (*Ufd1-like*) | #24700 (#1)  #104713 (#2) |
| *CDC45* | *CDC45L* | #41084 (#1)  #20705 (#2) |
| *SEPT5* | *Septin 4* (*Sep4*) | #7742 (#1)  #109398 (#2) |
| *TBX1* | *Optomotor-blind-related-gene-1* (*org-1*) | #37656 (#1)  #104393 (#2) |
| *GNB1L* | *CG13192* | #32157 (#1)  #110748 (#2) |
| *TXNRD2* | *Thioredoxin reductase-1, -2* (*Trxr-1, -2*) | #16768 (#1)  #50336 (#2) |
| *DGCR8* | *partner of drosha* (*pasha*) | #40118 (#1)  #107445 (#2) |
| *TRMT2A* | *CG3808* | #34713 (#1)  #108653 (#2) |
| *C22orf25* | *Transport and Golgi organization 2 (Tango2*) | #18175 (#1) |
| *ZDHHC8* | *Zinc finger DHHC-type containing 8* (*Zdhhc8*) | #5520 (#1)  #5521 (#2) |
| *DGCR6L* | *gonadal* (*gdl*) | #24453 (#1)  #23456 (#2) |
| *MED15* | *Mediator complex subunit 15* (*MED15*) | #21809 (#1) |
| *PI4KA* | *Phosphatidylinositol 4-kinase III α* (*PI4KIIIα*) | #15993 (#1)  #105614 (#2) |
| *SNAP29* | *Synaptosomal-associated protein 29 kDa* (*Snap29*) | #18173 (#1)  #107947 (#2) |
| *CRK* | *Crk oncogene* (*Crk*) | #19061 (#1)  #106598 (#2) |
| *AIFM3* | *CG4199* | #26424 (#1)  #106170 (#2) |
| *LZTR1* | *CG3711/Leucine zipper like transcription regulator 1* (*Lztr1*) | #11164 (#1) #13008 (#2) |
| *SLC7A4* | *CG13248* | #102635 (#1) |

Numbers in parentheses refer to the RNAi line numbers in Figure 1.
